# Elucidating Unknown Organofluorine in Municipal Wastewater: A Mass Balance Approach including Fluorinated Pharmaceuticals

**DOI:** 10.1021/acs.est.5c13161

**Published:** 2026-02-20

**Authors:** Pontus Larsson, Anna Kärrman, Leo W.Y. Yeung

**Affiliations:** Man-Technology-Environment (MTM) Research Centre, School of Science and Technology, 684587 Örebro University, Örebro SE-701 82, Sweden

**Keywords:** fluorine mass balance, extractable (organo)fluorine (EOF), multisorbent extraction, fluorinated pharmaceuticals, inorganic fluorinated compounds

## Abstract

Previous studies on per- and polyfluoroalkyl substances (PFAS) have indicated large amounts of unidentified organofluorine in municipal wastewater, raising concerns about their environmental impact. Here, a novel multisorbent solid phase extraction method was applied to municipal wastewater samples, followed by liquid chromatography–high-resolution mass spectrometry-based screening and a quantification workflow combining targeted analysis and combustion ion chromatography for fluorine mass balance analysis. Twenty-three highly fluorinated compounds (i.e., perfluoroalkyl acids and precursors) were identified and, apart from trifluoroacetic acid, quantified in the low- to sub-ppt range. In contrast, 30 low-fluorinated substances (i.e., active pharmaceutical ingredients, pesticides, and transformation products, including some previously unreported metabolites) were identified and quantified at concentrations up to 3 orders of magnitude higher. Despite their lower fluorine content (<30% by mass), these pharmaceuticals accounted for 28–42% of the extractable (organo)fluorine (EOF), with sitagliptin, bicalutamide, and celecoxib carboxylic acid being important drivers of the EOF. The inorganic fluoroanions hexafluorophosphate and tetrafluoroborate were coextracted and contributed 7–19% of the EOF. The multisorbent approach also captured polar cationic pharmaceuticals, substantially influencing the EOF composition. These findings highlight the complexity of fluorine mass balance in municipal wastewater and the need for advanced methods to uncover unidentified organofluorine.

## Graphical abstract

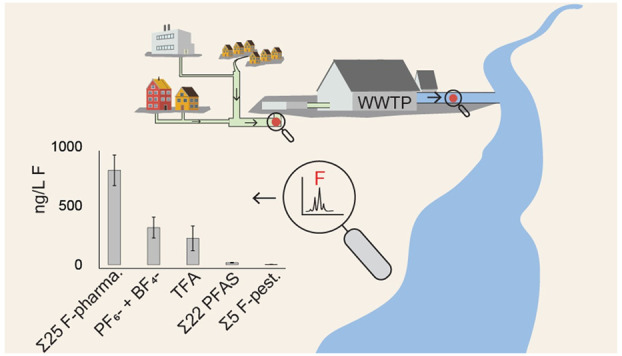

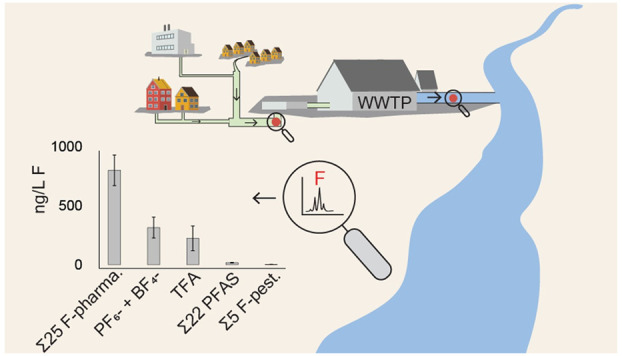

## 1 Introduction

Fluorine occurs naturally almost exclusively in inorganic forms,^1^ but rare examples of naturally occurring organofluorines exist—for instance, certain plants produce organic monofluorinated compounds like fluoroacetate. Conversely, synthetic organofluorine has been extensively manufactured since the 1940s. Due to the unique properties of fluorine, organofluorines have found diverse applications—e.g., in refrigerants,^2^ nonstick coatings,^3^ water- and oil-repellant products,^4^ pharmaceuticals,^5^ and agrochemicals.^6^ Per- and polyfluoroalkyl substances (PFAS), a subgroup of organofluorines, have gained increased interest due to their widespread occurrence in the environment and biota,^7,8^ as well as their adverse health effects on humans and wildlife.^9^ The chemical diversity within the PFAS subgroup is large. The total number of PFAS depends on the chemical definition and relevancy, but estimates range from ∼250 commercially relevant PFAS^10^ to over 7 million possible structures.^11^ Certain fluorinated pharmaceuticals and agrochemicals (e.g., fluoxetine, an antidepressant, and diflufenican, an herbicide), as well as refrigerants (e.g., R-134a), all of which contain at least one C–CF_3_ moetity, are considered PFAS according to a definition published in 2021 by the Organization for Economic Co-operation and Development (OECD).^12^ However, according to certain organizations and governments, the above-mentioned compounds fall outside the PFAS subgroup.^13^ Herein, the term *conventional PFAS* ^14^ is used to broadly refer to perfluoroalkyl acids (PFAA) and their precursors, while excluding compounds containing a C–CF_3_ moiety, such as certain pharmaceuticals and agrochemicals that may be considered precursors to the shortest-chain perfluorinated carboxylic acid (PFCA), trifluoroacetic acid (TFA).

The applicability of analytical methods suitable for determining PFAS as a group, e.g., assessing the total PFAS content in drinking water,^15^ has been discussed.^16,17^ Methods evaluated have included, among others: 1) high-resolution mass spectrometry (HRMS)-suspect screening and nontargeted workflows;^18^ 2) sample oxidation techniques to reveal unknown precursors;^19,20^ and 3) the use of combustion ion chromatography (CIC) to measure extractable (organo)fluorine (EOF/EF). The latter has previously been applied to various matrices, including wastewater.^21–23^ EOF methods are commonly combined with targeted liquid chromatography–tandem mass spectrometry (LC-MS/MS) analysis to compare the fraction of organofluorine quantified by targeted approaches with the broader, nonspecific coverage of the CIC method. This concept is often referred to as fluorine mass balance analysis^24^ and is used to estimate the amount of organofluorine unaccounted for in targeted analyses. Previous research^22^ has indicated that a large fraction of the EOF cannot be explained by conventional PFAS, resulting in a significant proportion of unknown EOF. Recently, it was shown that fluorinated pharmaceuticals can account for a substantial fraction of previously unidentified EOF in municipal wastewater and sludge.^14,25^ Previous fluorine mass balance studies of wastewater have exclusively employed a hydrophilic–lipophilic balance (HLB) or weak anion exchange (WAX)^22–24,26^ sorbent for the solid phase extraction (SPE) of wastewater samples, which intrinsically targets neutral and anionic species. The use of multifunctional sorbents and multilayered SPE has been investigated previously to enhance the retention and extraction recovery of a broader range of analytes^27–29^ but has yet to be applied to EOF methods. An assessment of a more inclusive extraction method for EOF analysis (e.g., multisorbent SPE) is, therefore, warranted.

This study introduces a workflow to improve the assessment of EOF in municipal wastewater by employing a multisorbent SPE to capture a broader spectrum of analytes. The workflow also enhances the quantification of organofluorine detected via HRMS screening, providing a more accurate evaluation of organofluorine loads entering and exiting wastewater treatment plants (WWTPs). By combining multisorbent SPE with advanced analytical techniques, this study elucidates the chemical composition of organofluorine and assesses the contributions of various fluorinated compounds, ranging from low-fluorinated substances, such as fluorinated pharmaceuticals and related compounds, to highly fluorinated conventional PFAS, to the overall organofluorine load in municipal wastewater.

## 2 Methods

### 2.1 Reagents and Standards

Details of the standards and reagents can be found in Supporting Information (SI).

### 2.2 Sample Collection and Preparation

Wastewater samples were collected from a WWTP located in Sweden. The plant served an average population equivalent (PE) of ∼160,000 and maintained a daily flow rate of around 40,000 m^3^/24 h during the time of sampling. Treatment of sewage involves a three-step process of mechanical, biological, and chemical treatment, without ozonation. Composite (24 h, flow-weighted) influent (*n* = 3) and effluent (*n* = 3) were sampled in paired sequence; i.e., effluent was collected 24 h later than influent to account for residence time in the treatment plant. Sampling was conducted three times, in May and September 2023 and December 2024, to represent different periods of the year; large seasonal variability in prescription rates of target pharmaceuticals was not expected (SI). Grab effluent samples (*n* = 6) were collected in May 2023 and pooled for use as quality control (QC) samples. Sampling was planned to avoid heavy rainfall prior to sampling. All samples were filtered through a 300 μm steel filter intended for another purpose outside of this study and were further decanted into 250 mL precleaned amber high-density polyethylene bottles (HDPE). The samples were stored at 4 °C until sample extraction, except for QC samples (grab) that were stored at −20 °C until the day of extraction. Further discussion on sampling and the potential impact of the filtering strategy is provided in the SI.

### 2.3 Sample Extraction

The extraction and preconcentration were performed using in-house packed multisorbent solid phase extraction cartridges, layered (bottom to top) with Oasis sorbents (Waters Corporation, Milford, MA, USA): strong cation exchange (MCX), WAX, and HLB. Each layer consisted of 150 mg of sorbent with a 30 μm particle size, separated by Oasis frits. Glass wool (20 mg) was placed on top of the packing bed to minimize and delay the clogging of the sorbent. A visual illustration and additional information on the multisorbent cartridge are provided in SI Figure S1. The solvent selection was adopted from the literature, including a wash step to remove free fluoride^22,24^ and an elution procedure.^26,30^

Triplicate extractions (each using 200–250 mL) of composite influent and effluent wastewater samples were processed together with two to three procedural blanks and one QC sample per extraction batch. Before sample loading, pH was adjusted to 3 using glacial acetic acid. Cartridges were preconditioned by sequentially passing 10 mL each of 2% ammonium hydroxide in methanol, followed by methanol and ultrapure water. After the sample was loaded at a flow rate of <1 drop/second, free fluoride was removed from the sorbent by an additional washing step consisting of 20 mL 0.01% ammonium hydroxide in ultrapure water, followed by 30 mL of ultrapure water and 4 mL of 25 mM ammonium acetate buffer (pH 4). The cartridges were dried under vacuum for 1 h. Elution was performed by 4 mL of methanol, followed by 6 mL of 2% ammonium hydroxide in methanol. The eluate was concentrated to a volume of approximately 0.2 mL under nitrogen before methanol was added for adjustment to a final volume of 0.5 mL. Further information can be found in SI.

Triplicate extractions were also carried out using the WAX-EOF protocol, following previously published methods^31^ for comparison with multisorbent extraction.

#### 2.3.1 Chemical Analysis

The sample extracts underwent a series of analyses, including EOF-CIC, LC-HRMS screening, and targeted analyses. A schematic overview of the analytical workflow is provided in Figure 1.

**Figure 1:**
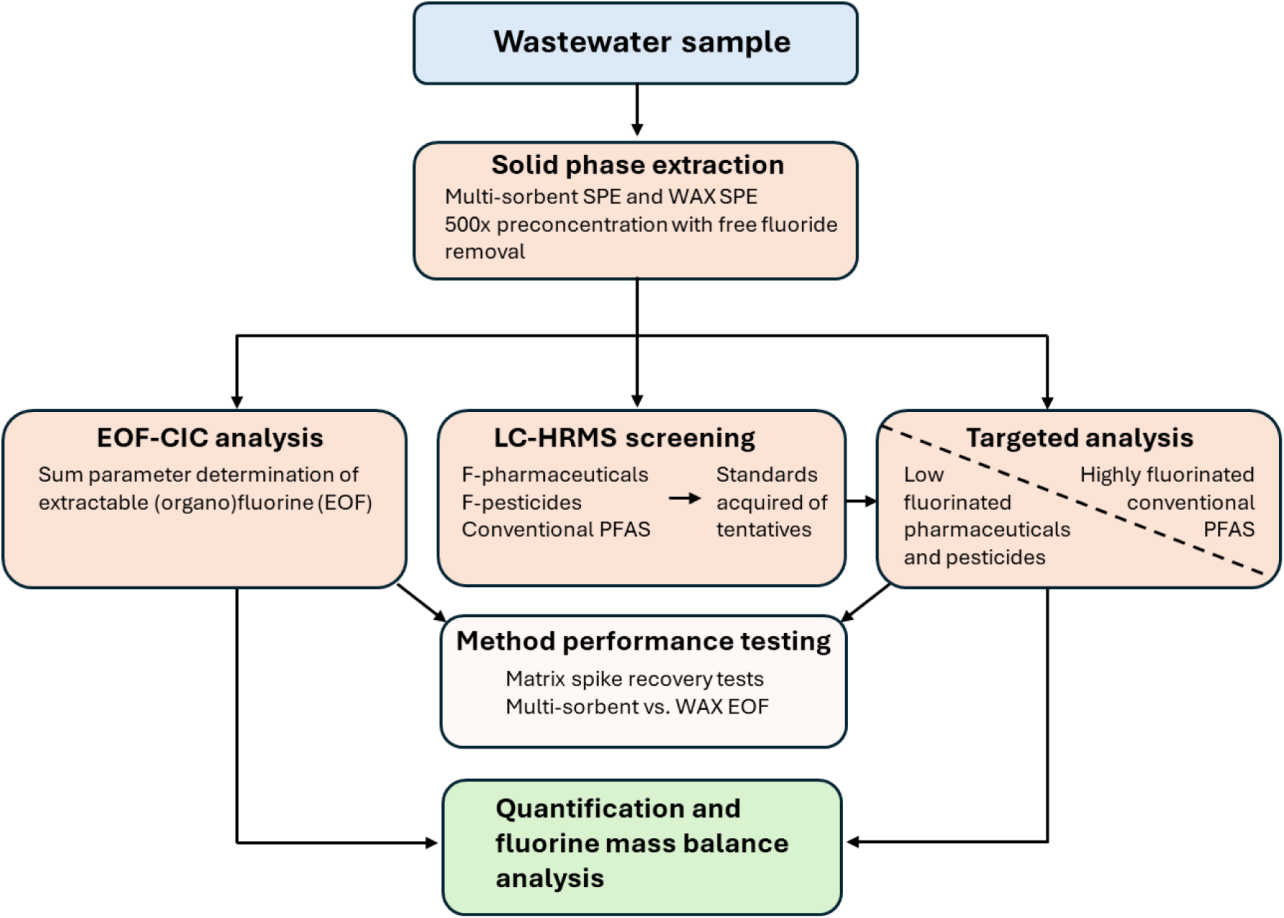
Overview of the analytical workflow used in this study for the assessment of organofluorine in wastewater.

#### 2.3.2 LC-HRMS Screening

High-resolution accurate mass (HRAM) data were acquired using an LC (Acquity UPLC, Waters Corporation, Milford, USA) coupled to a quadrupole time-of-flight mass spectrometer (G2-XS, Waters Corporation, Milford, USA), operated in electrospray negative ionization (ESI−) and electrospray positive ionization (ESI+) modes in separate injections. The data were obtained using data-independent acquisition (MS^e^), rather than a data-dependent acquisition mode, to improve retrospective analysis capability. A low collision energy (3 eV) scan (denoted MS1) was followed by a high energy scan (denoted MS2), with a collision energy ramp from 10 to 35 eV. The scan range was *m*/*z* 50–1200 with a scan time of 0.2 s. Within-run mass calibration was performed by infusion of a solution containing leucine enkephalin every 10 s. UNIFI 1.9.4 (Waters Corporation, Milford, MA, USA) was used for data acquisition, lock-mass correction, and further peak processing. Details are referred to SI including Table S4.

Suspect screening using HRAM data was performed via curated lists of fluorinated pharmaceuticals and pesticides, including metabolites and transformation products, as well as conventional PFAS. Further information on suspect lists is provided in the SI. Feature prioritization was done by applying mass filtering at 5 ppm mass error and an isotope match filter (isotope match intensity RMS percent: 40 and isotope match *m*/*z* RMS ppm: 10). Further data curation included MS2 spectral matching using the MassBank online repository,^32^ literature, and *in silico* fragmentation tools (UNIFI 1.9.4, MetFrag^33^), together with manual peak curation, e.g., fragment ion search. Reference materials for tentative suspects were acquired based on confidence in the identification and availability. Typically, the confidence level (CL) of identification reached CL2-3 ^34^ (i.e., most tentative candidates with MS2 fragment(s) matching with a library or diagnostic *in silico* fragments).

#### 2.3.3 Quantitative Analysis

Quantification was conducted using LC-MS/MS, which targeted 105 compounds over four separate methods and supercritical fluid chromatography (SFC)-MS/MS, where nine compounds were targeted in a single run. Further details are provided in SI, including Tables S1–S3, S5–S6.

##### 2.3.3.1 LC-MS/MS Conventional PFAS Analysis

The targeted analysis of conventional PFAS using LC-MS/MS has been described in detail and validated previously.^35^ In short, an Acquity UPLC was coupled to an XEVO TQ-S tandem mass spectrometer (UPLC-MS/MS, Waters Corporation, Milford, MA, USA), operating in multiple reaction monitoring (MRM).

##### 2.3.3.2 LC-MS/MS Analysis of Fluorinated Pharmaceuticals, Pesticides, and Related Compounds

The LC-HRMS screening served as a basis for the selection of most target analytes in LC-MS/MS quantification of fluorinated pharmaceuticals, pesticides, and related compounds. The instrumental analysis of fluorinated pharmaceuticals, pesticides, and related compounds employed two separate LC-MS/MS methods using the same system as for conventional PFAS analysis, using MRM in both electrospray positive ionization (ESI+) and ESI– ionization modes.

##### 2.3.3.3 SFC-MS/MS Analysis of Polar Anions

For compounds exhibiting little to no retention on a reversed-phase liquid chromatography system, a supercritical fluid chromatograph (SFC) coupled to a tandem mass spectrometer (Acquity Ultra Performance Convergence Chromatograph, XEVO TQ-S micro, Waters Corporation, Milford, MA, USA) was used. Operating in ESI– mode and utilizing both MRM and single ion recording (SIR) for acquisition, five ultrashort-chain PFAAs (C2–C3 perfluorocarboxylic acids (PFCAs), C1–C3 perfluorosulfonic acids (PFSAs)), three inorganic fluorinated anions (hexafluorophosphate [PF_6_^–^]), tetrafluoroborate [BF_4_^–^] and bis­(fluorosulfonyl)­imide [FSI]), and one pharmaceutical (5-fluorouracil) were separated and analyzed in a single run.

#### 2.3.4 Combustion Ion Chromatography for Extractable Organofluorine Analysis (CIC-EOF)

The CIC-EOF has been described elsewhere.^24^ In brief, an aliquot (0.1 mL) of the sample extract was combusted at 1000–1050 °C, where the formed hydrogen fluoride (HF) was adsorbed in ultrapure water, separated, and quantified as fluoride in an ion chromatograph. An autosampler and combustion module (Analytik Jena, Jena, Germany) were used together with a 920 Absorber Module and 930 Compact IC Flex (Metrohm, Herisau, Switzerland). Further details on method evaluation are provided in SI and elsewhere.^36^

#### 2.3.5 Quality Assurance and Quality Control

For each extraction batch, two or three procedural blanks were extracted, together with samples, to monitor background contamination. As fluoride is expected to be present at orders of magnitude higher concentration than organofluorine in wastewater, separation of fluoride during sample preparation is needed. A QC effluent sample (250 mL) was fortified with 5 mg L^–1^ sodium fluoride and was extracted in each extraction batch to monitor the repeatability and wash efficiency of free fluoride. The wash efficiency was evaluated with the QC sample with (*n* = 7) and without (*n* = 3) spiking of sodium fluoride. The accuracy of the sodium fluoride-fortified sample (*n* = 7) compared to nonfortified (*n* = 3) was 107%. Ultrapure water (250 mL) fortified with 5 mg L^–1^ sodium fluoride (*n* = 3) displayed a 99.99% removal efficiency, and at 0.3 mg L^–1^ (*n* = 1), the removal efficiency was 99.97%. Total fluorine (TF) of influent and effluent samples from 2023 was between 0.2 and 0.3 mg L^–1^ and was used as a proxy for the upper-bound concentration of inorganic fluorides. The multisorbent-EOF method (interday) repeatability, expressed as the coefficient of variance (CV) of measured EOF in QC samples (*n* = 7) extracted on five separate occasions, was 32%, comparable with the performance of WAX-EOF methods.^31,37^ For CIC analysis, an empty combustion boat was injected before every standard and sample and prebaked in the oven to minimize carryover. Every batch of samples was quantified using a calibration curve of PFOA at a concentration that ranged from 100 to 2000 ng/mL F equivalents, displaying a coefficient of determination (*R* ^2^) of a minimum of 0.99. The EOF concentrations in samples were processed after subtracting the procedural blank signal.

For quantification of target analytes, mass-labeled standards were added to the sample extract after extraction and quantified using internal standard calibration. Calibration curves were run, consisting of 10 calibration points, with the lowest calibration limit ranging from 0.005 to 10 ng mL^–1^ and an upper limit range of 50–200 ng mL^–1^, depending on the analyte. Further details, including a detailed description of the quantification methods and method quantification limits (MQLs), can be found in SI Table S8. Interday repeatability (CV) of analytes above MQL and quantitatively reported (*n* = 32) in the QC effluent samples (*n* = 4) ranged from 3 to 32% (median 15%).

To assess both analyte extraction recovery and instrumental matrix effects, a spike recovery test in the influent and effluent matrix was conducted with all target compounds, including the compounds newly identified via the LC-HRMS screening. Approximately 90% of all test compounds were extracted with a recovery within 60–130%. This range was used as the recovery criterion for the quantitative reporting herein. Further data on extraction recoveries, including comparisons to WAX SPE, quantification accuracies, and instrumental matrix effects, can be found in SI, including Tables S11−S13.

## 3 Results and Discussion

### 3.1 LC-HRMS-Based Screening

The focus of the LC-HRMS screening was to investigate the occurrence of low-fluorinated compounds (i.e., a fluorine mass fraction of ∼40% or less) not commonly targeted in PFAS analyses. Full-scan data were matched against a curated suspect list containing mainly fluorinated pharmaceuticals and metabolites. These matches were manually interrogated and constituted the basis for the acquisition of standards. Six compounds detected via suspect screening only reached a tentative identification level; these are summarized in Table 1 and are briefly discussed below. An expanded discussion on the HRMS data of tentatively detected compounds can be found in the SI and Table S12.

**Table 1:** List of Tentatively Identified Compounds in WWTP Influent and Effluent^a^

Compound name	Neutral formula	Confidence level^b^	Estimated effluent concentration range (ng L^–1^)
**MeFBSAA**	C_7_H_6_F_9_NO_4_S	CL 2b	1–10
**1:2** **H-PFESA**	C_3_F_6_H_2_SO_4_	CL 2b	1–10
**Bicalutamide TP-445**	C_18_H_14_F_4_N_2_O_5_S_2_	CL 3	1–10
**Bicalutamide TP-525A**	C_18_H_14_F_4_N_2_O_8_S_2_	CL 2b	10–100
**Bicalutamide TP-525B**	C_18_H_14_F_4_N_2_O_8_S_2_	CL 2b	1–10
**Sitagliptin TP-449**	C_18_H_17_F_6_N_5_O_2_	CL 3	100–1000

a Estimated concentrations were semiquantified by external calibration using surrogates. Further information is referred to SI Table S11.

b Confidence levels are reported based on Schymanski et al.^34^

A feature assigned as a hydrogen-substituted perfluoroether sulfonic acid (likely 1:2 H-PFESA) was tentatively detected in both the influent and effluent. This compound has previously been tentatively identified in wastewater (China^38^ and Belgium^39^) and recently in a large European screening study.^40^ No other hydrogen-substituted perfluoroether sulfonic acids (PFESAs) were detected in the present study, and no chlorine-substituted PFESAs were detected in the targeted analysis that may be precursors to certain H-PFESAs. 1:2 H-PFESA was identified at CL 2b and semiquantified to 1–10 ng L^–1^. Moreover, *N*-methylperfluorobutane sulfonamidoacetic acid (MeFBSAA) was identified at CL 2b and semiquantified to 1–10 ng L^–1^. Perfluoroalkane sulfonamidoacetic acids have been reported as intermediate environmental transformation products.^41^

Three hydroxylated bicalutamide transformation products (TP-446, TP-525A, and TP-525B) were identified at CL 3 and CL 2b, with two suspected to be sulfated at the hydroxyl group (TP-525A, TP-525B). Both hydroxy-bicalutamide and sulfated hydroxy-bicalutamide were reported as metabolites detected in bovine urine and feces.^42^ Recently, hydroxy-bicalutamide was reported for the first time in the environment and tentatively identified (CL 2b) in sewage sludge from Sweden^14^ with corresponding fragments of TP-525A (sulfated hydroxy) reported herein (SI, Table S10). The concentration of bicalutamide transformation products in the present study was estimated to be between 1 and 100 ng L^–1^ each, which is lower than the bicalutamide parent (see Table 2). Comparable peak areas of transformation products were observed in both effluent and influent, demonstrating their resistance to degradation.

**Table 2:** List of Identified Fluorinated Compounds Quantified with Reference Materials via Targeted LC-MS/MS or SFC-MS/MS Methods^a^ ^b^

		Compound name	Contain C–CF_3_?	Molecular formula	Concentration influent (ng L^–1^ min–max)	Concentration effluent (ng L^–1^ min–max)
**Low fluorinated**	**Pharmaceutical (active ingredient)**	Atorvastatin	No	C_33_H_35_FN_2_O_5_	ND–100*^a^	ND–<MQL*
		Bendroflumethiazide	Yes	C_15_H_14_F_3_N_3_O_4_S_2_	<MQL	<MQL–0.8
		Efavirenz	Yes	C_14_H_9_ClF_3_NO_2_	1–10*	1–10*
		Flucloxacillin	No	C_19_H_17_ClFN_3_O_5_S	<MQL–100*	1–100*
		Ticagrelor	No	C_23_H_28_F_2_N_6_O_4_S	7.8–11	<MQL–5.4
		Rufinamide	No	C_10_H_8_F_2_N_4_O	4.8–9.6	<MQL–1.0
		Fluoxetine	Yes	C_17_H_18_F_3_NO	<MQL–54	5.7–15
		Celecoxib	Yes	C_17_H_14_F_3_N_3_O_2_S	22–70	38–47
		Flecainide	Yes	C_17_H_20_F_6_N_2_O_3_	32–42	27–47
		Ezetimibe	No	C_24_H_21_F_2_NO_3_	61–79	30–34
		Citalopram	No	C_20_H_21_FN_2_O	100–340	140–210
		Fluconazole	No	C_13_H_12_F_2_N_6_O	120–140	68–110
		Bicalutamide	Yes	C_18_H_14_F_4_N_2_O_4_S	220–410	210–370
		Emtricitabine	No	C_8_H_10_FN_3_O_3_S	360–430	38–45
		Rosuvastatin	No	C_22_H_28_FN_3_O_6_S	430–500	240–520
		Sitagliptin	Yes	C_16_H_15_F_6_N_5_O	1400–2200	780–1300
	**Pharmaceutical metabolite**	Atorvastatin 4-hydroxy	No	C_33_H_35_FN_2_O_6_	ND–100*	ND−<MQL
		Efavirenz 8-hydroxy	Yes	C_14_H_9_ClF_3_NO_3_	10−100*	<MQL–10*
		Enzalutamide carboxylic acid	Yes	C_20_H_13_F_4_N_3_O_3_S	23–45	23–54
		Sitagliptin N-sulfate	Yes	C_16_H_15_F_6_N_5_O_4_S	10–100*	10–100*
		Rufinamide carboxylic acid	No	C_10_H_7_F_2_N_3_O_2_	36–57	28–49
		Ticagrelor deshydroxyethoxy	No	C_21_H_24_F_2_N_6_O_3_S	38–72	<MQL
		Flecainide meta-O-dealkylated	Yes	C_15_H_19_F_3_N_2_O_3_	45–74	8.0–19
		Citalopram desmethyl	No	C_19_H_19_FN_2_O	53–260	110–230
		Celecoxib carboxylic acid	Yes	C_17_H_12_F_3_N_3_O_4_S	240–340	350–510
	**Pesticide and metabolite**	Fluxapyroxad	No	C_18_H_12_F_5_N_3_O	0.2–0.7	0.2–0.6
		Fipronil-sulfone	Yes	C_12_H_4_Cl_2_F_6_N_4_O_2_S	0.6–1.1	0.8–0.9
		Fludioxonil	No	C_12_H_6_F_2_N_2_O_2_	<MQL–10*	<MQL–10*
		Fluopyram	Yes	C_16_H_11_ClF_6_N_2_O	1.1–4.9	0.9–2.1
		Fipronil	Yes	C_12_H_4_Cl_2_F_6_N_4_OS	2.5–7.7	2.8–8.0
**Highly fluorinated** ^c^	**Conventional PFAS**	Bis(trifluoromethylsulfonyl)imide	No	C_2_F_6_NO_4_S_2_H	0.2–1	0.2–0.5
		ΣPerfluoralkyl carboxylic acids (*n* = 7)	Yes	C_*n*_F_(2*n*+1)_CO_2_H (*n* = 2, 4, 5–10)	9.4–18	12–20
		ΣPerfluoroalkyl sulfonic acids (*n* = 4)	Yes	C_*n*_F_(2*n*+1)_SO_3_H (*n* = 1, 4, 6, 8)	6.7–12	7.7–10
		ΣPerfluoroalkyl acid precursors (*n* = 10)	Yes	C_*n*_F_(2*n*+1)_R (*n* = 4, 5, 6, 8)	4.1–7.8	1.6–3.6
		Trifluoroacetic acid	Yes	CF_3_CO_2_H	100–1000*	100–1000*
	**Inorganic fluoroanions**	Hexafluorophosphate	No	PF_6_^–^	63–190	70–170
		Tetrafluoroborate	No	BF_4_^–^	10–1000*	10–1000*

a HRMS spectrum of low-fluorinated compounds identified in this study is provided in SI.

b Compounds with a concentration denoted with * are reported on an order of magnitude basis, either due to low extraction recovery or inadequate accuracy in the quantification.

c Highly fluorinated was defined as a molecule having a fluorine mass fraction of ∼40% or higher (i.e., PFAAs and PFAA precursors) while low-fluorinated falls below this value (see SI Figure S2 and Tisler et al.^40^). All pharmaceuticals, pesticides, and related compounds included in this study maintained a fluorine mass fraction of <30%.

d Not detected (ND).

In total, 30 fluorinated compounds were tentatively identified in the screening with all compounds, except two, being low fluorinated. As a result of the screening, 24 standards were acquired. An additional four standards were acquired based on their occurrence in Swedish WWTPs, prescription rate, and, in one case, the detection of a metabolite in HRMS screening, which prompted further investigation of the parent pharmaceutical. Furthermore, an additional 10 low-fluorinated standards were available in-house and were incorporated into the quantification workflow and used for method performance testing.

### 3.2 Occurrence of Fluorinated Compounds in WWTP Influent and Effluent

Targeted LC- or SFC-MS/MS analyses confirmed the presence of 23 conventional PFAS and two inorganic fluoroanions. In addition, for compounds initially detected in the LC-HRMS screening, a follow-up targeted analysis was performed when reference standards were available. This approach reduced the risk of false negatives and provided quantitative data for selected low-fluorinated compounds. In total, 55 fluorinated compounds were detected above the MQL across all methods. These included 23 conventional PFAS previously mentioned, 30 low-fluorinated organofluorines (16 active pharmaceutical ingredients, nine metabolites, four pesticide active ingredients, and one pesticide transformation product), and two inorganic fluorinated compounds. The quantitative occurrence and distribution of these compounds in influent and effluent are presented in this section and summarized in Table 2.

#### 3.2.1 Fluorinated Pharmaceuticals, Pesticides, and Related Compounds

Twelve fluorinated pharmaceuticals and metabolites containing a C–CF_3_ moiety accounted for ∼65% of the sum concentration of all the low-fluorinated compounds in both influent and effluent. The average sum concentrations were found to be 2800 ng L^–1^ in the influent and 2000 ng L^–1^ in the effluent. Thirteen pharmaceuticals containing only moieties where a carbon atom is covalently bound to one or two fluorine atoms had an average sum concentration of 1700 ng L^–1^ in the influent and 1000 ng L^–1^ in the effluent. The group containing the C–CF_3_ moiety is of particular interest, as some compounds have been shown to degrade to TFA through biological or chemical transformation.^43–46^ TFA concentrations were shown to increase in fresh waters^47,48^ and understanding potential sources is of importance. C–CF_3_ containing pesticides have been suggested as a source of TFA in surface water from agriculturally intensive areas,^49^ while in a wastewater treatment system, it remains unclear how much C–CF_3_ pharmaceuticals contribute to the TFA concentration. Likely, this depends on the treatment technology employed.^44^ Five fluorinated pesticides, including one transformation product (fipronil sulfone), accounted for <0.5% of the sum concentration of low-fluorinated compounds.

Sitagliptin, an antidiabetic pharmaceutical, was the most predominant organofluorine detected in this study, with an average concentration of 2000 ng L^–1^ in influent and 1100 ng L^–1^ in effluent, corresponding to 43% and 35%, respectively, of the sum concentration of low-fluorinated compounds. Previously, sitagliptin has been measured in wastewater effluent from Hungary (588–1058 ng L−1 ^50^ ) and Austria (117 ng L−1 ^51^ ). The relatively high concentration of sitagliptin in this study is not surprising, given its widespread use and daily administration. The concentration of sitagliptin parent in influent was predicted to be approximately 2500 ng L^–1^, derived via data from the Swedish National Board of Health and Welfare^52^ (see SI for detailed information). One metabolite of sitagliptin (N-sulfated) was quantified to a lower concentration range of 10–100 ng L^–1^. This is in agreement with the metabolism of sitagliptin, where approximately 80% of the administered dose is expected to be excreted unchanged.^53^

The concentration of bicalutamide, a pharmaceutical used for prostate cancer treatment, ranged between 220 and 410 ng L^–1^ in influent and 210–370 ng L^–1^ in effluent, comparable with a recent study,^40^ where the concentration of bicalutamide was reported as 353–627 ng L^–1^ in two WWTPs from Sweden. The detection of bicalutamide, together with the tentative detection of several transformation products, is of interest, as bicalutamide was identified as a top-prioritized pollutant in Swedish wastewaters,^54^ receiving the highest ecological score among 119 prioritized pollutants (∼15,000 total entries). Bicalutamide ranked second only to venlafaxine in the final combined scoring of ecological risks, environmental and human health hazards.^55^ However, due to limited information on the occurrence, fate, and ecological risk of bicalutamide transformation products, they were not included in the above-mentioned study.

Celecoxib, a nonsteroidal anti-inflammatory pharmaceutical, was detected both as its parent compound and as celecoxib carboxylic acid, a phase I metabolite. The concentration of the parent compound was quantified between 22 and 70 ng L^–1^ (influent) and 38–47 ng L^–1^ (effluent). A previous study reported concentrations in effluent between 17 and 49 ng L−1 ^56^ and 19 ng/g dw in sewage sludge from Sweden.^14^ In this study, celecoxib carboxylic acid was quantified at around 1 order of magnitude higher concentration than its parent compound: 240–340 ng L^–1^ (influent) and 350–510 ng L^–1^ (effluent). Celecoxib carboxylic acid was previously tentatively identified in wastewater.^56^ The higher measured concentration of the metabolite is expected, as it is extensively metabolized, with less than 3% excreted unchanged.^57^ Transformation primarily involves hydroxylation of the methyl phenyl group, followed by oxidation into carboxylic acid, which can further undergo glucuronidation. The increase in the concentration of the carboxylic metabolite observed in effluent may be due to cleavage of the glucuronide side chain or other conjugates occurring during wastewater treatment processes.^58^

Other notable pharmaceuticals detected were rosuvastatin (410 ng L^–1^), citalopram (180 ng L^–1^), fluconazole (92 ng L^–1^), and emtricitabine (40 ng L^–1^), listed with average effluent concentrations in parentheses. To the best of our knowledge, some of the fluorinated pharmaceuticals and metabolites identified in this study have not yet been reported in the environment or municipal wastewater, or the literature on their environmental occurrence is scarce. For example, carboxylic acid metabolites of enzalutamide (also referred to as M1) and rufinamide (also known as CGP 47292), for which no report on their environmental detection was found; rufinamide parent (100 μg L^–1^ reported in effluent from an epilepsy ward,^59^ but no other data on the environmental occurrence); and ticagrelor deshydroxyethoxy (also known as AR-C124910XX), which has only been tentatively identified in sewage sludge recently.^14^

Most detected fluorinated pesticides were fungicides: fludioxonil, fluopyram, and fluxapyroxad, with the exception of the insecticide fipronil; a compound that has been restricted in the European Union since 2013 for use in plant protection products.^60^ Fipronil is still marketed and sold as a veterinary drug product for pet flea and tick treatment, and a recent study^61^ estimated 20–40% of fipronil may enter the wastewater via this product category. To the best of our knowledge, this study is the first to report fluopyram and fluxapyroxad in municipal wastewater.

#### 3.2.2 Inorganic Fluorinated Anions

PF_6_^–^ and BF_4_^–^ were shown to be coextracted in WAX methods typically used for EOF analysis.^37^ Their inclusion in a fluorine mass balance context is therefore important to estimate amounts of wanted or unwanted coextracted inorganics. In the present study, PF_6_^–^ was measured at a concentration ranging from 63 to 190 ng L^–1^ (influent) and 70–170 ng L^–1^ (effluent). The quantification accuracy of BF_4_^–^ was not satisfactory due to significant matrix enhancement (∼400% in effluent, ∼200% in influent) and low extraction recovery (17% in effluent and 39% in influent); the concentration is therefore reported in the range of 10–1000 ng L^–1^. The presence of PF_6_^–^ and BF_4_^–^ in both influent and effluent in the present study, as well as recent prior detections in surface water^62^ and drinking water^37^ is notable. Neither anion appears particularly unstable hydrolytically nor readily degradable in wastewater. Aqueous solutions of lithium PF_6_^–^ are reported by the European Union Chemical Agency (ECHA) as hydrolytically unstable with a half-life of 0.05 min,^63^ degrading to F^–^ and PO_4_^3–^; i.e., the assumed end products following LiPF6 exposure or environmental release. Conversely, in the literature,^64^ LiPF_6_ was reported as hydrolytically stable in aqueous solutions, with no degradation occurring after 30 days at pH 7, and only <50% degradation at pH 0.5. Potassium salt of PF_6_^–^ was reported^65^ as stable in aqueous solution, even at pH >12 and <1. Insights into the stability of LiPF_6_ ^64^ showed that trace amounts of water in a solvent system (i.e., an electrolyte environment of a lithium-ion battery) can lead to rapid hydrolysis of PF_6_^–^, while in an aqueous solution where PF_6_^–^ is fully solvated by water, its hydrolytic stability is much greater. This may help explain recent environmental detections of PF_6_^–^ and underscores that further risk assessments are needed due to the apparent aqueous and environmental stability of the PF_6_^–^ anion.

#### 3.2.3 Conventional PFAS

Recovery of TFA was poor (10–22%) with the CIC-EOF method and is, therefore, reported separately from other conventional PFAS. Its concentration was estimated to be in the range of 100–1000 ng L^–1^ in both the influent and effluent. Excluding TFA, the sum of 22 detectable conventional PFAS was between 24 and 37 ng L^–1^ (influent) and 21–32 ng L^–1^ (effluent), which is lower than what has previously been reported in samples collected in 2016–2017 from Swedish WWTPs (Uppsala effluent ∑26PFAS 170 ng L^–1^;^66^ Stockholm and Borås effluent ∑73PFAS 59 and 163 ng L^–1^, respectively^22^). In the present study, C3 and C5–C10 PFCAs accounted for the majority of the conventional PFAS in both influent and effluent. Perfluoropropionic acid (PFPrA) accounted for 23% (influent) and 22% (effluent) of the conventional PFAS and was present at an average concentration of 6.8 ng L^–1^ and 5.9 ng L^–1^ in influent and effluent, respectively, followed by PFOA (2.5 ng L^–1^ influent; 2.7 ng L^–1^ effluent) and perfluorohexanoic acid (PFHxA) (1.7 ng L^–1^ influent, 2.7 ng L^–1^ effluent). PFSAs (C1, C4–C6, C8) were present at approximately half the concentration of ∑PFCAs and made up approximately 30% of the ∑conventional PFAS in both influent and effluent. Trifluoromethanesulfonic acid (TFMS), had the highest concentration at 3.5 ng L^–1^ in influent and 4.2 ng L^–1^ in effluent, followed by PFOS (average: 2.9 ng L^–1^ influent and 2.7 ng L^–1^ effluent). PFAA precursors made up an average of 25% of the ∑conventional PFAS concentration in influent (average ∑PFAA precursor 6.5 ng L^–1^) and ∼ 10% in effluent (average 2.3 ng L^–1^). Bis­(trifluoromethylsulfonyl)­imide (NTF_2_), associated with lithium-ion batteries,^67^ was also detected in both influent and effluent samples, with average concentrations of 0.5 ng L^–1^ (influent) and 0.4 ng L^–1^ (effluent).

### 3.3 Extractable (Organo)Fluorine Mass Balance

The EOF concentration ranged from 1300 to 3300 ng L^–1^ F (influent) and 1400–2300 ng L^–1^ F (effluent), which is comparable to earlier reported concentrations in Nordic WWTPs.^22^ Conversion of mass concentration of individual compounds to fluorine equivalent concentrations (presented as ng L^–1^ F) was performed for all compounds (details are referred to SI). An overview of the fluorine mass balance is presented in Figure 2, including fluorine equivalent concentrations (panel A), EOF concentration and composition (panel B), distribution of the fluorine mass fraction, and fluorine equivalent concentrations in the effluent (panel C).

**Figure 2:**
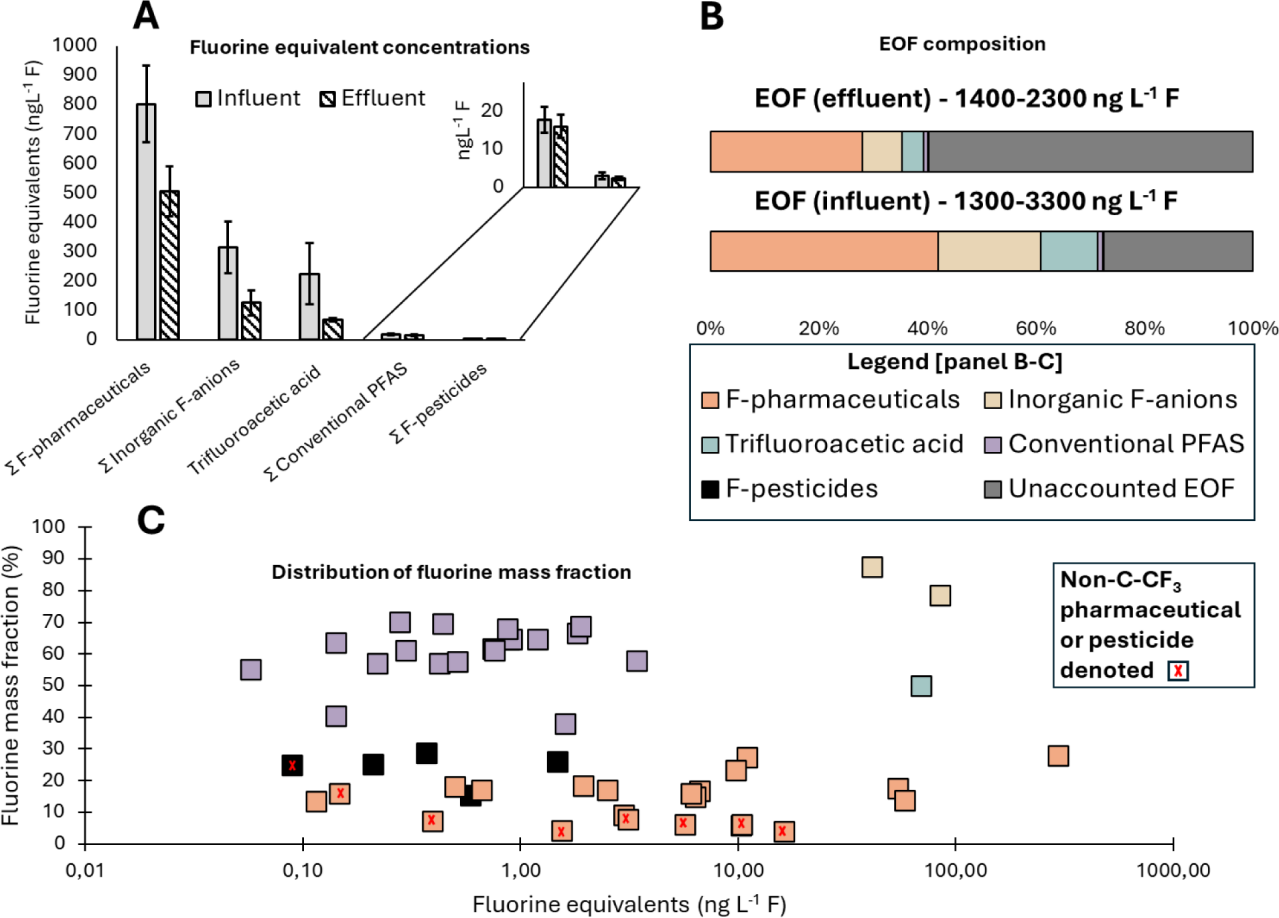
Composition and concentration of extractable (organo)fluorine compounds in wastewater influent and effluent. (A) Fluorine equivalent concentrations (expressed as ng L^–1^ F); error bars display the standard deviation of the average (*n* = 3) of triplicate or duplicate extractions. (B) Average concentrations of compound groups (converted to ng L^–1^ F) in relation to the sum value of extractable (organo)­fluorine, i.e., the EOF composition. (C) Distribution of quantified fluorinated compounds in effluent (converted to ng L^–1^ F) in relation to their fluorine mass fraction, calculated from the weight of fluorine atom(s) divided by the compounds’ molecular weight. Note: concentrations are not extraction recovery-corrected.

As shown in Figure 2, panels B and C, low-fluorinated organofluorine accounted for the majority of the EOF in influent. Most contained the substructure R-CF_3_, where R was a ring structure (e.g., aryl-CF_3_). This is in agreement with a recent study^68^ where fluorine nuclear magnetic resonance (F-NMR) spectroscopy was used to identify aryl-CF_3_-containing compounds as the major organofluorine associated with samples from WWTPs. In the present study, fluorinated pharmaceuticals, including their metabolites, had the greatest sum of fluorine equivalents, making up, on average, 42% (influent) and 28% (effluent) of the EOF. Within this group, active pharmaceutical ingredients made up a larger proportion; 37% in influent and 23% in effluent, with metabolites thereof accounting for 5% in both influent and effluent. Fluorinated pesticides accounted for less than 0.2% of the EOF, indicating that they are not important drivers. TFA made up a large proportion of the EOF, accounting for 11% of the influent and 4% in effluent. Excluding TFA, conventional PFAS accounted for 1% in both the influent and effluent. This is lower than what has been reported^22^ earlier Swedish effluent (average 14%), which may indicate a lower proportion of the EOF is explained by conventional PFAS in recent years. BF_4_^–^ and PF_6_^–^ together made up 19% and 7% of the EOF in influent and effluent, respectively, demonstrating the importance of inorganic fluoroanions in a fluorine mass balance context. In addition, due to the poor recovery of TFA and BF_4_^–^, it introduces a bias in the results that must be considered. Extraction recovery was greater in influent (TFA 22%, BF_4_^–^ 39%) than effluent (TFA 10%, BF_4_^–^ 17%); thus, TFA and BF_4_^–^ concentrations in the extracts are not representative of the water sample and further lead to a higher fraction of the TFA and BF_4_^–^ explaining the EOF in influent compared to effluent.

The most significant pharmaceuticals and related compounds contributing to the EOF were sitagliptin, followed by bicalutamide and celecoxib carboxylic acid, together accounting for 22–39% in influent and 19–26% in effluent. Sitagliptin alone accounted for an average of 29% in the influent and 16% in the effluent. In municipal wastewater from the United States,^25^ sitagliptin accounted for 9–100% of the EOF in influent and 10–45% in effluent, and in suspended particulate matter from German rivers,^69^ up to 94% of ∑_8_Organofluorine (detected via nontarget screening) was attributed to sitagliptin.

A large proportion of EOF still remained unidentified (influent, 28%; effluent, 60%). Semiquantification of tentatively identified organofluorine (Table 1) could not explain the majority of the unidentified EOF. While total oxidizable precursor assay experiments indicated the presence of unknown PFAA precursors—i.e., the concentration of C4–C10 PFAAs increased by a factor of 2–10× following oxidation—they only accounted for a minor proportion (≤2%) of the EOF mass balance (see the SI for more information). Nonetheless, due to a larger percentage of influent EOF known to consist mostly of pharmaceuticals and metabolites, it suggests that a large proportion of the unknown EOF in effluent could be their transformation products. The presence of unknown pharmaceutical-related transformation products is further indicated, considering that certain pharmaceuticals detected in this study are known to be extensively metabolized (e.g., flecainide^70^ and celecoxib^57^), while only part of the assumed excreted dose could be accounted for in the measured metabolite (flecainide meta-O-dealkylated and celecoxib carboxylic acid) herein. Transformation of pharmaceuticals and metabolites into unknown species during sample storage may explain part of the unidentified EOF. For example, 14–17% of 128 pharmaceuticals and personal care products were reported^71^ as unstable under the same storage conditions as this study. Phase II metabolites may be deconjugated into their respective parents or phase I metabolites during storage, or parent pharmaceuticals and metabolites may degrade into unknown species.^58^

Considering the removal efficiency of sodium fluoride in ultrapure water and an effluent matrix, the contribution of free fluoride to the EOF was considered minimal. Based on the removal efficiency in ultrapure water (99.97–99.99%), the upper-bound contribution of free fluoride was assessed to be 2–6% of the EOF. However, the actual contribution was likely lower, considering the sodium fluoride spike tests in effluent showed no significant influence on the EOF concentration at a 5 mg L^–1^ fluoride spike level. Nonetheless, the potential presence of inorganic fluorinated compounds or complexes other than PF_6_^–^ and BF_4_^–^, e.g., other hexa- or tetrafluoroanions such as hexafluoroarsenate (AsF_6_^–^) or tetrafluoroaluminate (AlF_4_^–^), should be considered. AsF_6_^–^ was recently identified in multiple European municipal wastewaters.^40^ In the current study, AsF_6_^–^ was not part of the targeted analysis, and the LC-HRMS screening method employed is likely not adequate for detection of this class of very polar compounds due to the expected lack of retention on a reversed-phase column.

### 3.4 Approach to Assess Organofluorine in Municipal Wastewater

A key objective of this study was to establish a quantitative workflow for organofluorine compounds identified through LC-HRMS screening, enabling a more accurate assessment of their concentrations. This was accomplished by developing targeted LC-MS/MS methods for newly identified compounds and validating extraction recovery in matrix-matched spike experiments. Out of all 114 test compounds, 104 (effluent) and 102 (influent) were extracted within a satisfactory recovery range of 60–130%, with the 25th to 75th quartile recovery ranging from 73 to 91% in influent and 89–99% in effluent. In contrast, the 24 compounds detected via LC-HRMS screening displayed a recovery from 40 to 109% in influent and 28–101% in effluent. Excluding atorvastatin and 4-hydroxy atorvastatin, recovery was 58–109% and 61–101% in influent and effluent, respectively. Due to the low fluorine mass fraction of atorvastatin and its hydroxylated metabolite (∼3%), a higher recovery would not impact the fluorine mass balance significantly. Spike and accuracy tests enabled quantitative reporting for most of the newly identified compounds; however, due to the limited number of mass-labeled internal standards in the targeted workflow and poor recovery for some, fully quantitative results were omitted for six out of the 24 compounds detected via LC-HRMS screening.

The spike experiments, together with the replicate extraction and EOF measurement using WAX-EOF, also enabled a comparison between multisorbent-EOF and single-sorbent-EOF. EOF concentration of multisorbent-EOF was, on average, 40–50% higher than WAX-EOF in influent and effluent (see SI Figure S3). In the spike tests, both methods displayed satisfactory recovery for most neutral and anionic compounds, and neither method effectively recovered the polar and lowest-molecular-weight compounds tested, though slightly better recovery was seen for multisorbent-EOF. For example, 5-fluorouracil, a small and polar pharmaceutical, TFA, and BF_4_^–^ displayed poor recovery (<1–39%) in multisorbent-EOF. Spike tests also revealed that PF_6_^–^, BF_4_^–^, and FSI (not detected in this study) recovery was between 17 and 101% (effluent) and 39–82% (influent) for multisorbent-EOF, while for WAX-EOF, the recovery was 6–85% (effluent) and 53–77% (influent). This further emphasizes the importance of inorganic compounds in a fluorine mass balance context and the need for further method optimization for true EOF determination.

Differences between the methods were observed for cationic pharmaceuticals and metabolites: WAX-EOF showed poor recovery, whereas multisorbent-EOF was effective for most. For eight cationic pharmaceuticals that maintained a net positive charge at pH 3 (the pH used for sample extraction), the 25th to 75th quartile recovery range was 79–95% and 4–66% in effluent, and 90–95% and 1–51% in influent, for multisorbent-EOF and WAX-EOF, respectively. Poor recovery of polar and cationic compounds for WAX is expected, as the sorbent is a mixed-mode lipophilic-hydrophilic (divinylbenzene and *N*-vinylpyrrolidone) copolymer modified with basic piperazinyl groups designed to retain anionic and neutral compounds. MCX, used in the bottom layer of the multisorbent, contains the same copolymer backbone but is modified with sulfonate groups that promote cation exchange retention, leading to an improved recovery of cations. Notably, the basic pharmaceutical sitagliptin, and the most prominent organofluorine in this study, and the largest contributor to the multisorbent-EOF mass balance showed poor recovery (≤1%) with WAX-EOF. These results demonstrate that EOF composition and concentration may vary depending on extraction method and extraction parameters used.

Furthermore, the overall extraction procedure and methodological framework applied within this study may be adopted for other aqueous matrices, such as surface or drinking water. Arising from the larger sorbent amount, one practical disadvantage of using the multisorbent approach is the increased back pressure of the cartridge, which may limit its application for samples with higher particle content.

## 4 Environmental Implications and Future Needs

Previous studies have shown that wastewater treatment plants release organofluorine compounds into the environment, many of which remain unidentified.^22,23^ Understanding their sources and quantifying the unidentified fraction are crucial to reducing human and environmental exposure. Persistent organofluorine may be released directly as stable compounds (e.g., PFAAs) or formed indirectly from certain substructures (e.g., C–CF_3_), which can degrade to TFA. This study advanced our knowledge by identifying low-fluorinated compounds, primarily pharmaceuticals and metabolites, as major contributors to EOF in wastewater. This is highlighted by several low-fluorinated compounds previously not quantified in municipal wastewater or the environment. Importantly, many of these compounds contained moieties with the potential to partly transform into TFA, whose concentrations in freshwater have increased rapidly in recent years.^47,48,72^ Our results provide a clue to potential sources of TFA in the environment, but a better understanding of the fate and transformation of C–CF_3_ compounds in wastewater treatment systems is critical for accurate assessment.

In this study, conventional PFAS were not significant contributors to the EOF mass balance. Thus, the use of EOF-CIC for assessing PFAS totality in wastewater needs careful consideration for its purpose, given the high mass of fluorinated pharmaceuticals, including monofluorinated, entering municipal wastewater. Instead, improved strategies are needed, e.g., more specific sum determination of persistent organofluorine species. For example, Zweigle et al. (2024) introduced the concept of fractionation of low-fluorinated organofluorine and highly fluorinated PFAA precursors via oxidative conversion. With further development of this concept, EOF measurement of wastewater could enable a selective determination of *persistent EOF* (e.g., sum parameter determination of all hydroxyl-radical-stable perfluoroalkyls). Furthermore, a multisorbent SPE method enables extraction of a broader range of anionic, neutral, and cationic compounds, providing greater nonspecific coverage of the method. Improved sample cleanup methods that separate inorganic fluorides, including inorganic fluorinated substances (e.g., via initial hydrolysis of PF_6_^–^ ^73^) while quantitatively recovering small, polar organofluorines (e.g., TFA), would enable more unbiased and true EOF determination. These improvements could enable more accurate monitoring of persistent organofluorine in wastewater.

## Supplementary Material





## Notes

Information on chemicals and materials; sample preparation; instrumental analysis; fluorine equivalent concentrations; total oxidizable precursor assay; quality control; influent and effluent concentrations; sitagliptin-predicted influent concentrations; LC-HRMS screening; method performance testing (PDF)
Information on suspect lists; LC-HRMS spectrum library of low-fluorinated compounds­(XLSX)
