# Supplementary material for: Elucidating Unknown Organofluorine in Municipal Wastewater: A Mass Balance Approach including Fluorinated Pharmaceuticals
Source: Environ Sci Technol. 2026 Feb 20;60(8):6623–34. doi: 10.1021/acs.est.5c13161 (PMC12961926; doi:10.1021/acs.est.5c13161)
Supplement: Supplementary file 1 [file es5c13161_si_001.pdf]

Supporting information

Elucidating unknown organofluorine in municipal wastewater: A mass balance approach including fluorinated pharmaceuticals

Pontus Larsson, Anna Kärrman, Leo W.Y. Yeung*

Man-Technology-Environment (MTM) Research Centre, School of Science and Technology, Örebro University, Sweden, SE-701 82

* Corresponding author: leo.yeung@oru.se

Table of contents

Chemicals and materials ... 2

Table S1. List of standards ... 2

Sample preparation ... 3

Figure S1. Schematic description of multi-sorbent SPE cartridge ... 4

Instrumental analysis ... 4

Table S2. Chromatographic gradient settings for LC/SFC methods ... 5

Table S3. MS source settings ... 5

Table S4. LC and MS source settings for LC-HRMS analysis ... 6

Table S5. List of compound specific MS source settings – low fluorinated ... 6

Table S6. Compound specific MS source settings ... 7

Fluorine equivalent concentration conversion ... 9

Figure S2. Fluorine mass fraction distribution of compounds ... 10

Total oxidizable precursor assay ... 11

Table S7. Concentration of PFAAs following oxidative conversion ... 11

Quality control ... 11

Table S8. Method quantification limits ... 12

Influent and effluent concentrations ... 15

Table S9. Concentration of target analytes ... 15

Sitagliptin predicted influent concentrations ... 18

LC-HRMS screening ... 18

Table S10. List of tentatively identified compounds. ... 21

Method performance testing ... 22

Table S11. Quantification of low-fluorinated compounds in influent and effluent ... 22

Table S12. Extraction recoveries of multi-sorbent SPE and WAX-SPE ... 24

Table S13. Matrix effect of multi-sorbent SPE and WAX-SPE ... 26

Figure S3. Concentration of EOF measured by multisorbent SPE and WAX-SPE ... 29

Chemicals and materials

Methanol (LC-MS grade), Acetonitrile (LC-MS grade), 25% ammonium hydroxide (analytical reagent grade) and sodium hydroxide (≥ 99%) and formic acid (LC-MS grade) were bought from Fisher Scientific (Pittsburgh, PA, USA). Acetic acid was bought from Merck (Darmstadt, Germany), Sodium bicarbonate/Sodium carbonate concentrate (for 20x dilution), potassium persulfate (reagent grade, ≥ 99%), and ammonium acetate (LC-MS grade) was purchased from Sigma-Aldrich (St Luis, MO, USA). Ultrapure water (MilliQ water; 18.2 MΩ·cm) was purified from a Millipore system. Ammonium hydroxide (LC-MS grade) and ammonium formate (LC-MS grade) were bought from Honeywell Fluka (Charlotte, NC, USA).

Stock standard solutions were diluted into working standard solutions of 200-2000 ng mL^{-1}, in either methanol or acetonitrile. Materials provided as salt from supplier were first dissolved into methanol at 1 mg mL^{-1} or lower, followed by further serial dilution into working standard solution of 200-2000 ng mL^{-1}. Standard solutions were stored at -20°C, except standards of perfluoroalkyl acids and precursors, that were stored in 4°C. Most native and isotopically labelled PFAS standards were purchased from Wellington Laboratories (Guelph, Canada), others included PFECHS (Chiron – Trondheim, Norway); FMeSI (Thermo Scientific, Waltham, MA, USA); FEtSI and FBuSI (Tokyo Chemical Industry, Tokyo, Japan); and FBSA, FHxSA (Apollo Scientific, Manchester, UK). A list of other fluorinated compounds (e.g., pharmaceuticals) and their respective suppliers can be found in table S1. A full list of target analytes can be found in tables S5 and S6.

Table S1. List of native and isotopically standards of low-fluorinated (pharmaceuticals, metabolites, pesticides, bisphenol AF) and inorganic fluorinated compounds.

Name	CAS no.	Supplier	Form	Purity
Rosuvastatin calcium	147098-20-2	Medchemexpress	10 mM*1 mL (DMSO)	>99%
Citalopram hydrobromide	59729-32-7	Medchemexpress	10 mM*1 mL (DMSO)	>99%
Efavirenz	154598-52-4	Medchemexpress	10 mM*1 mL (DMSO)	>99%
Ezetimibe	163222-33-1	Medchemexpress	10 mM*1 mL (DMSO)	>99%
Atorvastatin hemicalcium	134523-03-8	Medchemexpress	10 mM*1 mL (DMSO)	>99%
Ticagrelor	274693-27-5	Medchemexpress	10 mM*1 mL (DMSO)	>99%
Sitagliptin phosphate monohydrate	654671-77-9	Medchemexpress	10 mM*1 mL (water)	>99%
Bicalutamide	90357-06-5	Medchemexpress	10 mM*1 mL (DMSO)	>99%
Fluconazole	86386-73-4	Medchemexpress	10 mM*1 mL (DMSO)	>99%
Flecainide acetate	54143-56-5	Medchemexpress	10 mM*1 mL (DMSO)	>99%
Flucloxacillin sodium	1847-24-1	Medchemexpress	10 mM*1 mL (DMSO)	≥98.0%
Fluoxetine hydrochloride	56296-78-7	Merck/Cerilliant	1.0 mg/mL (MeOH)	>99%
Bendroflumethiazide	73-48-3	Sigma-Aldrich	Salt	>99%
Celecoxib	169590-42-5	Sigma-Aldrich	Salt	>99%
Ciprofloxacin	85721-33-1	Fluka	Salt	≥98%
Emtricitabine	143491-57-0	Tokyo Chemical Industry	Salt	>98.0%
Rufinamide	106308-44-5	Tokyo Chemical Industry	Salt	>98.0%
5-fluorouracil	51-21-8	Thermo Scientific	Salt	99%
Norfluoxetine oxalate	107674-50-0	Merck/Cerilliant	1.0 mg/mL (MeOH)	>99%
rac 8-Hydroxy Efavirenz	205754-32-1	Toronto Research Chemicals	Salt	>95%
rac Desmethyl citalopram hydrochloride	85118-27-0	aablocks	Salt	≥98%
Celecoxib carboxylic acid	170571-01-4	Toronto Research Chemicals	Salt	>95%
4-Hydroxy atorvastatin disodium	1276537-18-8	Toronto Research Chemicals	Salt	>95%

deshydroxyethoxy ticagrelor	220347-05-7	Chemscene	Salt	≥95%
Enzalutamide carboxyic acid	1242137-15-0	Medchemexpress	Salt	98%
Sitagliptin N-sulfate sodium	2470126-25-9	Toronto Research Chemicals	Salt	>95%
Rufinamide carboxylic acid	166196-11-8	Arctom Scientific	Salt	98%
Flecainide Meta-O-dealkylated	83526-33-4	Toronto Research Chemicals	Salt	>95%
Fipronil	120068-37-3	HPC standards	100 µg/ml (ACN)	
Fipronil-sulfone	120068-36-2	HPC standards	10.0 µg/ml (ACN)	
Benzovindiflupyr	1072957-71-1	HPC standards	100 µg/ml (ACN)	
Fluopyram	658066-35-4	HPC standards	100 µg/ml (ACN)	
Fluxapyroxad	907204-31-3	HPC standards	100 µg/ml (ACN)	
Flufenacet	142459-58-3	HPC standards	100 µg/ml (ACN)	
Fludioxonil	131341-86-1	HPC standards	100 µg/ml (ACN)	
Diflufenican	83164-33-4	Sigma-Aldrich	Salt	≥95%
Fluroxipyr	69377-81-7	Sigma-Aldrich	Salt	≥98%
Bisphenol AF	1478-61-1	Ambeed	Salt	98%
Potassium hexafluorophosphate	17084-13-8	Thermo Scientific	Salt	99%
Potassium tetrafluoroborate	14075-53-7	Thermo Scientific	Salt	99%
Potassium bis(fluorosulfonyl)imide	14984-76-0	Toronto Research Chemicals	Salt	>95%
Rosuvastatin-d3 sodium	1279031-70-7	Medchemexpress	Salt	>98%
Citalopram-d6 Oxalate	1246819-94-2	Toronto Research Chemicals	Salt	>95%
Fluoxetine-d6 oxalate	NA	Merck/Cerilliant	0.1 mg/mL (MeOH)	>98%

Sample preparation

Monthly pharmaceutical prescription rates were considered when planning the sampling campaigns but were deemed to not influence the study outcome substantially. For example, the Swedish monthly prescription rates of the antibiotic flucloxacillin varied a maximum of 21% (i.e., the percent difference between the highest and lowest prescribed month) in the years 2023 and 2024. Similarly, the antidepressants fluoxetine and citalopram/escitalopram varied to a maximum of 7% and 19%, respectively[30]. Nonetheless, this study did not attempt to assess any seasonal variation of EOF in wastewater.

Samples were filtered through a steel filter (300 µm) for another purpose outside of this study. Because this is not a commonly used mesh size for this type of analysis, possible implications may be considered. Because no smaller filter mesh size was used (e.g., 0.2-2 µm), concentrations reported in this study should not be interpreted as, what is conventionally considered[1, 2], dissolved phase concentration. It is further possible that certain compounds may be preferably portioned to particles which may be filtered out. For example, Aro et al., 2021 [2] examined the PFAS concentrations and fluorine mass balance of municipal wastewater from Nordic countries and reported that, on average, more than 90% of the sum of 73 PFAS partitioned into the (sub 0.7 µm) aqueous phase. Based on this evaluation, the overall impact on quantified PFAS concentrations is considered minimal.

Multi-sorbent SPE cartridges were in-lab from Oasis SPE products (Waters Corporation, Milford, USA). Oasis MCX cartridges (6cc, 150 mg, 30 um) were layered with sorbent material from Oasis HLB and WAX (30 um), separated by frits (Oasis). An illustration can be found in figure S1.

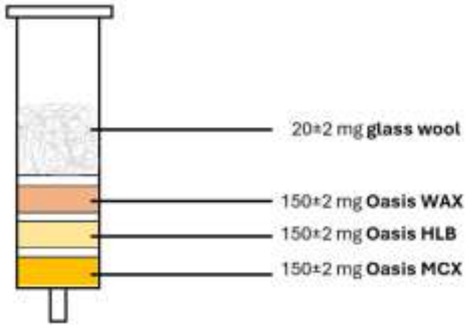

Figure S1. Schematic description of multi-sorbent SPE cartridge.

Single layer (WAX) solid phase extraction:
Composite influent and effluent samples (200-250 mL) were extracted in triplicates, with two or three procedural blanks and one QC sample in each batch. pH was adjusted to 3 with glacial acetic acid. Conditioning of cartridges were performed with 4 mL 0.1% ammonium hydroxide in methanol, 4 mL methanol and 4 mL ultrapure water. Sample loading was carried out at a rate of <1 drop/second and the cartridges were washed with 20 mL 0.01% ammonium hydroxide in ultrapure water followed by 30 mL ultrapure water and 4 mL 25 mM ammonium acetate buffer (pH 4). To dry the sorbent bed, vacuum was applied for one hour. Elution was performed with 4 mL 0.1% ammonium hydroxide in methanol, that was further concentrated under nitrogen to a volume of 0.2 mL and adjusted to 0.5 mL using methanol.

Extract preparation for instrumental analysis
For both multi-sorbent and WAX extraction, the methanolic extract (0.5 mL) was divided for extractable organofluorine analysis (0.12 mL) and LC-HRMS analysis (0.1 mL methanolic extract and 0.1 mL of 10 mM ammonium acetate). The remaining extract was stored at -20°C. A third extract split of 0.1 mL extract and 0.1 mL of 10 mM ammonium acetate was prepared, with internal standards added (2-5 ng), intended for the targeted quantification workflow. Extracts were centrifuged at 6000 rpm for 10 minutes to settle potential precipitates and were transferred to new sample vials on a per-sample basis in case particles would interfere with the instrumental injection. In these cases, extraction blank samples were also transferred.

Instrumental analysis

LC-HRMS-screening
The mobile phase selection was based on a previously published LC-HRMS screening method [3] with minor modifications. For both ionization modes, a mix of ultrapure water/methanol 95:5 as mobile phase A and methanol for mobile phase B was used. In negative mode, 5 mM ammonium acetate + 0.1% ammonium hydroxide were added to the mobile phases; while in positive ionization mode, 5 mM ammonium formate + 0.1% formic acid was used. An Acquity Premier BEH C18 column (2.1 mm × 100 mm; 1.7 µm; Waters Corporation, Milford, MA, USA) was kept at 50°C, and used for the chromatographic separation, with a 5 µL injection volume in both ionization modes.

A data-independent acquisition (DIA) was selected over a data-dependent acquisition (DDA) approach primarily due to having an improved ability to retrospectively analyze the data. Furthermore, the workflow in this study was better suited to DIA, because the compound lists (e.g., for use in DDA inclusion lists) were not always complete before injection, and the manual data interrogation workflow used in this study more naturally aligned with a DIA approach.

Because the workflow relied heavily on confirming HRMS suspects with reference materials, the need for consistently high-quality MS^2 spectra that was also considered less critical.

LC-MS/MS conventional PFAS analysis
The extract was injected twice with minor changes to MS settings (see SI table S3). For chromatographic separation, an Acquity Premier BEH C18 column (2.1 mm × 100 mm, 1.7 µm; Waters Corporation Milford, MA, USA) was used with mobile phases 2 mM ammonium acetate in 30:70 MeOH/ultrapure water (A) and 2 mM ammonium acetate in methanol (B). For most PFAS, two MRM transitions were used except for some where only one transition could be used.

LC-MS/MS analysis of fluorinated pharmaceuticals, pesticides and related compounds
The instrumental analysis of fluorinated pharmaceuticals, pesticides and related compounds employed two separate LC-MS/MS methods, using the same system as described in the section above, with settings such as mobile phases, analytical column and gradient based on the conditions from the LC-HRMS screening but with minor modifications. In ESI-, 0.1% ammonium hydroxide and 5 mM ammonium acetate in 10% methanol/90% ultrapure water (A) and methanol (B) with 5 µl injection volume. In ESI+, 0.1% formic acid and 5 mM ammonium formate, with the same solvent compositions as negative mode and with 1 µl injection volume.

SFC-MS/MS
Super-/subcritical separation was done using a Torus DIOL (3 mm x 100mm x 1.7 µm, Waters Corporation, Milford, MA, USA) analytical column, maintained at 40°C, with mobile phases CO_2 (A) and 0.1% ammonium hydroxide in methanol (B). The applied chromatographic method has been reported previously [4], but MS settings were modified. A make-up solvent containing methanol was continuously pumped at 0.1 mL/min and the automated backpressure regulator was set at 1500 psi.

Combustion ion chromatography
For chromatographic separation, a Metrosep A Supp5-150/4 analytical column and a carbonate buffer (3.2 mM sodium carbonate and 1 mM sodium bicarbonate) was used as the mobile phase.

Table S2. Chromatographic gradient settings for LC/SFC methods A, B- and B+ and method C.

**Target_A (LC-MS/MS)**			**Target_B- (LC-MS/MS) Target_B+ (LC-MS/MS)**			**Target_C (SFC-MS/MS)**		
**time** (min)	**Mobile phase A** (%)	**Mobile phase B** (%)	**time** (min)	**Mobile phase A** (%)	**Mobile phase B** (%)	**time** (min)	**Mobile phase A** (%)	**Mobile phase B** (%)
0.00	99	1	0.00	99	1	0.00	95	5
0.57	99	1	0.10	99	1	6.00	60	40
13.00	0	100	12.00	0	100	7.00	60	40
14.00	0	100	15.00	0	100	7.10	95	5
14.20	99	1	15.10	99	1	8.00	95	5
17.00	99	1	18.00	99	1			

Table S3. MS source settings for all targeted analysis. A1 refers to target PFAS analysis. A2 refers to second target PFAS analysis with source settings optimized for diPAPs. Method B- and B+ refers to analysis of pharmaceuticals, pesticides and related compounds as well as cationic conventional PFAS (B+). C refers to SFC-MS/MS method of polar anionic compounds.

	A1 (LC-MS/MS)	A2 (LC-MS/MS)	B-, B+ (LC-MS/MS)	C (SFC-MS/MS)
**Source temperature**	150°C	150°C	150°C	120
**Desolvation temp**	400°C	200°C	400°C	450°C
**Desolvation gas flow**	800 L/h	800 L/h	800 L/h	600 L/h
**Cone gas flow**	150 L/h	150 L/h	150 L/h	1 L/h
**Capillary voltage**	0.70 kV	2.90 kV	2.50 kV	0.8 kV

Table S4. LC and MS source settings for LC-HRMS analysis.

**time** (min)	**Mobile phase A** (%)	**Mobile phase B** (%)	**MS settings**	**Neg**	**Pos**
0.00	99	1	**Source temperature**	150°C	150°C
0.10	99	1	**Desolvation temp**	400°C	400°C
12.00	0	100	**Desolvation gas flow**	800 L/h	800 L/h
15.00	0	100	**Cone gas flow**	50 L/h	50 L/h
15.01	99	1	**Capillary voltage**	2.5 kV	3.0 kV
18.00	99	1	**Cone voltage**	40	30 V

Analyte tuning procedure

To determine precursor ion to fragment ion mass transitions and optimal source settings for newly discovered compounds, reference solutions in methanol or acetonitrile at 10-100 ng/mL were infused via an automatic syringe pump to the MS and manually tuned for each compound, using a combined flow of the respective mobile phases of 0.1 mL/min (LC) and an infusion rate of 0.005-0.01 mL/min. For all compounds, the adducts $[M-H]^-$ (ESI-) and $[M+H]^+$ (ESI+) were monitored for the precursor ion and between two and six MRM transitions were monitored for each component. While generally the most abundant MRM transition was selected for quantification, in some cases, a lower abundant MRM transition was selected to increase the upper linear range of the method. For compounds ionizable in both positive and negative mode, the selection of polarity was based on detection limits and degree of matrix effect. Optimized source settings and MRM specific settings (i.e., collision and cone voltage) can be found in table S5-S6.

Table S5. List of compound specific MS source settings (cone voltage and collision energies) of pharmaceuticals, metabolites, pesticides, bisphenol AF, for corresponding MRM channels, and internal standard used for quantification.

Name	Source polarity	Precursor/fragment ion (m/z) (quantification)	Cone (V)	Col (eV)	Precursor/fragment ion (m/z) (qualification)	Cone (V)	Col (eV)	Internal standard influent	Internal standard effluent
Rosuvastatin	Negative	480.3/418	20	14	480.3/298	20	30	d3-rosuvastatin	d3-rosuvastatin
Rosuvastatin (positive)**	Positive	482.2/258	20	31	482.2/300	20	35	d3-rosuvastatin	d3-rosuvastatin
Citalopram*	Positive	325.2/116	20	25	325.2/262	20	20	d6-citalopram	d6-citalopram
Efavirenz	Negative	314.0/244	20	15	314.0/230	20	12	d3-rosuvastatin	d3-rosuvastatin
Ezetimibe	Negative	408.1/271	20	15	408.1/175	20	30	d3-rosuvastatin	d3-rosuvastatin
Atorvastatin	Negative	557.2/278	20	45	557.2/397.2	20	30	d3-rosuvastatin	d3-rosuvastatin
Ticagrelor*	Positive	523.2/293	20	30	523.2/127	20	50	d3-rosuvastatin	d3-rosuvastatin

Sitagliptin	Positive	408.1/174	30	25	408.1/235	30	18	d3-rosuvastatin+d6-citalopram	d3-rosuvastatin+d6-citalopram
Bicalutamide*	Negative	429.1/173	20	25	429.1/185	20	40	d3-rosuvastatin	d3-rosuvastatin
Fluconazole	Positive	307.1/238	20	12	307.1/220	20	15	d3-rosuvastatin+d6-citalopram	d3-rosuvastatin+d6-citalopram
Flecainide*	Negative	413.2/353	20	15	413.2/373	20	8	d3-rosuvastatin	d3-rosuvastatin
Flecainide (positive)**	Positive	415.2/301	20	32	415.2/398	20	22	NA	NA
Flucloxacillin	Positive	354.1/196	80	25	354.1/238	80	15	d3-rosuvastatin	d3-rosuvastatin
Fluoxetine	Positive	310.2/44	20	10	310.2/148	20	8	d6-fluoxetine	d6-fluoxetine
Bendroflumethiazide	Negative	420.0/289	25	23	420.0/328	20	25	d3-rosuvastatin	d3-rosuvastatin
5-fluorouracil	Negative	129/42	25	12	129/86	25	16	d3-rosuvastatin	d3-rosuvastatin
8-hydroxy efavirenz	Negative	330.0/210	20	18	330.0/258	20	18	d3-rosuvastatin	d3-rosuvastatin
Desmethyl citalopram*	Positive	311.2/116	20	25	311.2/262	20	15	d6-citalopram	d6-citalopram
Celecoxib carboxylic acid*	Negative	410.0/282	20	25	410.0/262	20	30	d3-rosuvastatin	d3-rosuvastatin
4-hydroxy atorvastatin	Negative	573.2/413.4	20	30	573.2/469.4	20	25	d3-rosuvastatin	d3-rosuvastatin
Deshydroxyethoxy ticagrelor	Positive	479.2/153	20	35	479.2/127	20	50	d3-rosuvastatin	d3-rosuvastatin
Seproxetine	Positive	296.2/134	20	6	296.2/30	20	10	d6-fluoxetine	d6-fluoxetine
Enzalutamide carboxylic acid	Negative	450.1/406.1	20	15	450.1/253.1	20	35	d3-rosuvastatin	d3-rosuvastatin
Sitagliptin N-sulfate	Negative	486.1/96	20	25	486.1/252	20	30	d3-rosuvastatin	d3-rosuvastatin
Celecoxib	Positive	382.1/362	20	29	382.1/282	20	35	d3-rosuvastatin	d3-rosuvastatin
Ciprofloxacin	Positive	332.2/314	20	20	332.2/288	20	17	13C$_3$,15N-ciprofloxacin	13C$_3$,15N-ciprofloxacin
Emtricitabine	Positive	248.0/130	20	10	248.0/101	20	20	d6-citalopram	d3-rosuvastatin
Rufinamide	Positive	239.1/127.1	20	20	239.1/144	20	10	d6-citalopram	d3-rosuvastatin
Rufinamide carboxylic acid	Positive	240.1/127.1	20	23	240.1/222.1	20	10	d3-rosuvastatin	d3-rosuvastatin
Flecainide Meta-O-dealkylated	Positive	333.1/219	70	28	333.1/316	70	18	d3-rosuvastatin+d6-citalopram	d3-rosuvastatin+d6-citalopram
Diflufenican	Positive	395.2/266	20	35	395.2/246	20	32	d3-rosuvastatin	d3-rosuvastatin
Fluroxipyr	Negative	252.9/195	10	10	252.9/233	10	10	d3-rosuvastatin	d3-rosuvastatin
Fipronil	Negative	434.9/330	25	15	434.9/399	25	10	d3-rosuvastatin	d3-rosuvastatin
Fipronil-sulfone	Negative	450.9/282	25	25	450.9/415	25	15	d3-rosuvastatin	d3-rosuvastatin
Benzovindiflupyr	Positive	398.1/342	25	18	398.1/378	25	15	d3-rosuvastatin	d3-rosuvastatin
Fluopyram	Positive	397.1/208	25	20	397.1/173	25	35	d6-citalopram	d3-rosuvastatin
Fluxapyroxad	Negative	380.1/91	50	35	380.1/248	50	22	d3-rosuvastatin	d3-rosuvastatin
Flufenacet	Positive	364.1/152	25	20	364.1/194	25	10	d6-citalopram	d3-rosuvastatin
Fludioxonil	Negative	247.0/180	25	30	247.0/169	25	30	d3-rosuvastatin	d3-rosuvastatin
Bisphenol AF	Negative	335.1/265	25	25	335.1/197	25	35	d3-rosuvastatin	d3-rosuvastatin

*non-optimal MRM transitions used to increase upper calibration range

** optimized but not selected for target method

Table S6. Compound specific MS source settings (cone voltage and collision energies) for conventional PFAS and inorganic fluorinated anions. for corresponding MRM channels and internal standard used for quantification.

Name	Precursor/fragment ion (m/z) (quantification)	Cone (V)	Col (eV)	Precursor/fragment ion (m/z) (qualification)	Cone (V)	Col (eV)	Internal standard

TFMS	149.12/79.91	20	15	149.12/98.95	20	5	$^{13}C_3$-PFBS
PFEtS	198.8/79.8	65	20	198.8/98.9	65	35	$^{13}C_3$-PFBS
PFPrS	249.0/80.0	70	25	249.0/99.0	70	30	$^{13}C_3$-PFBS
PFBS	298.9/98.9	20	26	298.9/79.96	20	26	$^{13}C_3$-PFBS
PFPeS	348.90/98.96	20	26	348.90/79.96	20	30	$^{18}O_2$-PFHxS
PFHxS	398.9/98.9	20	30	398.9/79.96	20	34	$^{18}O_2$-PFHxS
PFHpS	448.97/98.90	20	30	448.97/79.96	20	35	$^{13}C_4$-PFOS
PFOS	498.97/98.96	20	38	498.97/79.96	20	44	$^{13}C_4$-PFOS
PFNS	548.90/98.96	20	38	548.90/79.96	20	44	$^{13}C_4$-PFOS
PFDS	598.97/98.9	20	42	598.97/79.96	20	58	$^{13}C_4$-PFOS
PFUnDS	648.9/98.9	20	40	648.9/79.96	20	45	$^{13}C_4$-PFOS
PFDoDS	698.90/98.90	20	40	698.90/79.96	20	45	$^{13}C_4$-PFOS
PFTrDS	748.9/98.9	20	40	748.9/79.96	20	45	$^{13}C_4$-PFOS
TFA	112.9/68.96	26	10				$^{13}C_2$-TFA
PFPrA	162.97/118.9	20	10				$^{13}C_4$-PFBA
PFBA	212.97/169	20	11				$^{13}C_4$-PFBA
PFPeA	262.97/219	20	8				$^{13}C_3$-PFPeA
PFHxA	312.97/269	20	9	312.97/118.95	20	26	$^{13}C_2$-PFHxA
PFHpA	362.97/319	20	10	362.97/168.97	20	16	$^{13}C_4$-PFHpA
PFOA	412.97/369	20	10	412.97/168.97	20	18	$^{13}C_4$-PFOA
PFNA	462.99/419	20	12	462.99/219	20	18	$^{13}C_5$-PFNA
PFDA	512.97/469	20	11	512.97/219	20	18	$^{13}C_2$-PFDA
PFUnDA	562.97/519	20	12	562.97/268.99	20	18	$^{13}C_2$-PFUnDA
PFDoDA	612.97/569	34	14	612.97/168.96	40	22	$^{13}C_2$-PFDoDA
PFTrDA	662.9/619	20	14	662.9/168.96	20	26	$^{13}C_2$-PFDoDA
PFTDA	712.9/669	20	14	712.9/168.97	20	28	$^{13}C_2$-PFTDA
PFHxDA	812.9/769	30	15	812.9/168.96	42	32	$^{13}C_2$-PFHxDA
PFOcDA	912.9/869	36	15	912.9/168.96	36	36	$^{13}C_2$-PFHxDA
3:3 FTCA	240.9/116.98	10	30	240.9/176.98	10	8	$^{13}C_2$-6:2 FTUCA
5:3 FTCA	340.9/236.97	10	16	340.9/216.93	10	22	$^{13}C_2$-6:2 FTUCA
7:3 FTCA	440.9/316.93	12	14	440.9/336.89	12	20	$^{13}C_2$-8:2 FTUCA
6:2 FTUCA	356.9/292.91	10	18	356.9/242.95	10	36	$^{13}C_2$-6:2 FTUCA
8:2 FTUCA	456.9/392.94	10	18	456.9/342.88	10	38	$^{13}C_2$-8:2 FTUCA
10:2 FTUCA	556.84/492.82	8	16	556.84/242.94	8	38	$^{13}C_2$-10:2 FTUCA
4:2 FTSA	327/307	20	20	327/81	20	28	$^{13}C_2$-6:2 FTSA
6:2 FTSA	427/407	20	20	427/81	20	28	$^{13}C_2$-6:2 FTSA
8:2 FTSA	527/507	20	20	527/80	20	28	$^{13}C_2$-8:2 FTSA
10:2 FTSA	627/607	20	20	627/80	20	28	$^{13}C_2$-8:2 FTSA
6:2 diPAP	788.9/97	64	28	788.9/442.91	64	18	$^{13}C_4$-6:2 diPAP
8:2 diPAP	988.78/96.94	68	34	988.78/542.81	68	26	$^{13}C_4$-8:2 diPAP
6:2/8:2 diPAP	888.78/96.94	66	34	888.78/442.81	66	26	$^{13}C_4$-6:2 diPAP
SAmPAP	649.78/96.87	2	26	649.78/168.91	2	36	$^{13}C_2$-8:2 monoPAP
diSAmPAP	1202.65/525.87	92	46	1202.65/168.90	92	64	$^{13}C_2$-8:2 diPAP

PFHxPA	398.97/79	62	26				$^{13}C_4$-PFOA
PFOPA	499/79	62	30				$^{13}C_4$-PFOA
C6/C6 PFPiA	701/401	62	28				$^{13}C_2$-PFDoDA
C6/C8 PFPiA	801/401	24	28	801/501	24	28	$^{13}C_2$-PFTDA
C8/C8 PFPiA	901/501	24	28				$^{13}C_2$-PFTDA
FBSA	297.9/77.92	20	20	297.9/118.94	20	15	$^{13}C_4$-PFBS
FHxSA	397.9/77.92	30	26	397.9/168.94	30	28	$^{18}O_2$-PFHxS
FOSA	497.9/78	82	30	497.9/468.96	82	28	$^{13}C_8$-FOSA
FOSAA	555.84/497.82	60	26	555.84/418.85	60	24	$^{2}H_5$-Et-FOSAA
MeFOSAA	569.78/418.87	16	18	569.78/482.77	16	14	$^{2}H_5$-Et-FOSAA
EtFOSAA	583.84/418.84	18	20	583.84/525.88	18	20	$^{2}H_5$-Et-FOSAA
MeFBSA	311.97/111.93	14	16	311.97/218.95	14	16	$^{13}C_4$-PFBA
MeFHxSA	411.97/168.93			411.97/318.96			$^{2}H_3$ MeFOSA
MeFOSA	512.0/169	27	45				$^{2}H_3$ MeFOSA
EtFOSA	526.0/169	27	45				$^{2}H_5$ EtFOSA
ADONA	376.97/250.83	30	37	376.97/84.69	15	29	$^{13}C_3$-HFPO-DA
HFPO-DA	284.9/184.8	20	7	284.9/168.7	20	17	$^{13}C_3$-HFPO-DA
3,6-OPFHpA	201.0/84.9	10	15				$^{13}C_3$ HFPO-DA
PF4OPeA	229.0/84.9	10	10				$^{13}C_3$ HFPO-DA
PFEESA	314.9/134.9	14	23				$^{13}C_3$ PFBS
6:2 Cl-PFESA	530.90/350.98	58	24	530.90/83.03	58	24	$^{13}C_4$-PFOS
8:2 Cl-PFESA	630.90/450.98	58	24	630.90/83.03	58	24	$^{13}C_4$-PFOS
FMeSI	279.9/147	30	23	279.9/78	30	35	$^{13}C_3$ PFBS
FEtSI	379.9/197	30	25	379.9/78	30	35	$^{13}C_3$ PFBS
FBSI	579.9/297	30	30	479.9/78	30	40	$^{18}O_2$-PFHxS
PFECHS	460.84/98.88	2	26	460.84/380.94	2	24	$^{13}C_4$-PFOA
5:3 FTB*	414.1/58	20	35	414.1/104	20	35	NA
AP-FHxSA*	485.1/85	30	30	485.1/58	30	40	NA
TAmP-FHxSA*	499.1/60	20	35	499.1/73	20	35	NA
6:2 FTAB*	571.1/104	20	30	571.1/58	20	30	NA
FSI	179.9/97.0	20	25	179.9/99	20	25	$^{13}C_2$-TFA
Hexafluorophosphate**	144.96	25					$^{13}C_2$-TFA
Tetrafluoroborate**	87	25		86	25		$^{13}C_2$-TFA

*ESI+ mode
** Single ion recording mode

Fluorine equivalent concentration conversion

Measured mass concentrations (ng L^{-1}) of fluorinated compounds were converted to fluorine equivalent concentration (ng L^{-1} F) according to the following formula:

$$C_F = n_F * \frac{MW_{fluorine}}{MW_{analyte}} * C_{analyte}$$

Where C_F is the fluorine equivalent concentration (ng L^{-1} F), n_F is the number of fluorine atoms of the molecule, $MW_{Fluorine}$ is the molecular weight of fluorine and $MW_{analyte}$ is the molecular weight of the analyte and $C_{analyte}$ is the concentration (ng L^{-1}) of the analyte.

As internal standards were added after extraction, the concentrations determined via LC-MS/MS or SFC-MS/MS are directly comparable with the concentration determined via CIC, following conversion into ng L^{-1} as described below.

A visual representation of the fluorination degree of highly fluorinated conventional PFAS and low fluorinated compounds, in relation to their molecular weight, can be found in figure S2. Typically, fluorinated pharmaceuticals maintain a fluorine mass fraction of 30% or less. Some rare exceptions exist however, e.g., perfluorohexyloctane, a medication for dry eyes that received Unted States Food and Drug administration (US FDA) approval in 2023 [5], which has a fluorine mass fraction of 57%. Conventional PFAS and low fluorinated pharmaceuticals and pesticides exhibit distinct patterns of the fluorine mass fraction and a separation of the organofluorine based on this descriptor is possible. Conventional PFAS, such as perfluoroalkyl derivates, generally has a mass fraction of ~55±15%, where a lower fluorine mass fraction is seen for ultrashort chain compounds (TFMS 38%; NTF_2 41%; TFA 50%) and a higher mass fraction is seen for longer chain compounds (PFOS, 65%; PFOA 69%). The inorganic anions detected in this study, PF_6^- and BF_4^-, had the highest fluorine mass fraction, at 79% and 86%, respectively.

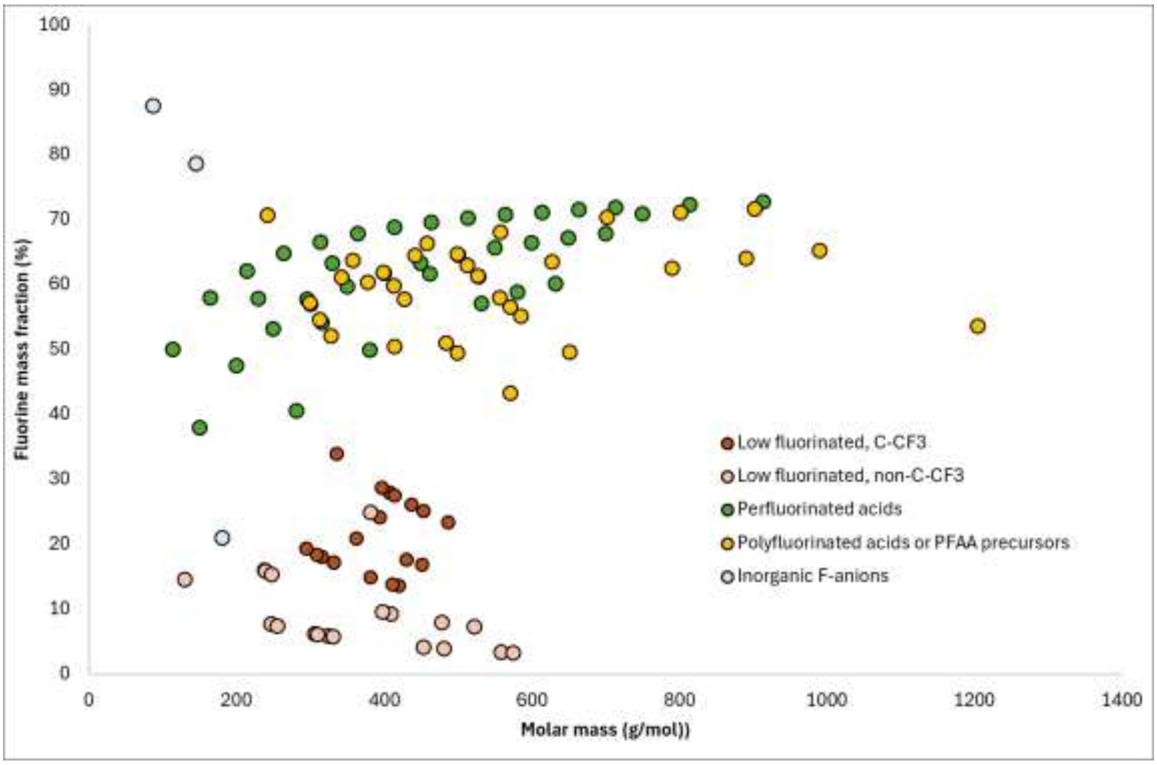

Figure S2. Fluorine mass fraction distribution of compounds that were part of the targeted method. Per- and polyfluorinated acids and PFAA precursors were denoted as highly fluorinated, with a fluorine mass fraction of ~40% or higher. Fluorinated pharmaceuticals, pesticides and related compounds and bisphenol AF were denoted as low fluorinated whilst having a fluorine mass fraction of <30% and 34%, respectively.

Total oxidizable precursor assay

Oxidative conversion of PFAA precursors was performed, following previously published protocols [6]. Briefly, triplicate or duplicate effluent and influent extracts were pooled to a total volume of 0.1 mL and spiked into a 50 mL polypropylene tube. Extracts were let to evaporate until approximately 0.02 mL was left and was amended with 0.8g potassium peroxydisulfate, 0.75 mL 10 N NaOH and was filled up to 50 mL with ultrapure water, equivalent to an oxidant and base concentration of 60 mM and 150 mM respectively. Procedural blanks were processed the same way as samples. Before heating the reaction for six hours in an 85°C water bath, 5 µL of 1 µg/mL M8-FOSA was added to monitor the oxidation efficiency of the reaction. pH was measured before and after the reaction to confirm a pH of >12 was maintained. After the reaction, samples were put on ice and stored at 4°C. Internal standards (2 ng) were added before samples were adjusted to pH 2 using formic acid and SPE (500 mg Oasis WAX, 30 µm) was performed as follows. Cartridges were conditioned by sequentially passing 10 mL 0.1% ammonium hydroxide in methanol, 10 mL methanol, 10 mL ultrapure water. After sample loading at a flow rate of approximately 1 mL/min, the cartridges were washed with 10 mL ultrapure water and 4 mL of 25 mM ammonium acetate in ultrapure water. Following drying under vacuum for one hour, the samples were eluted with 10 mL 0.1% ammonium hydroxide in methanol. Subsequently, samples were evaporated under a stream of nitrogen until 0.1 mL remained, centrifuged at for 10 minutes at 6000g, and transferred to a LC-vial. Finally, samples were diluted 1:1 with a solution of 10 mM ammonium acetate.

As quality control of the oxidative conversion, the transformation of spiked (5 ng) M8-FOSA to M8-PFOA was determined. The average yield in all samples was 102% (RSD 5%).

Table S7. Concentration of PFAAs following oxidative conversion, together with its increase in concentration compared to before oxidation. In some cases (e.g., PFBA), compounds could not be quantified before oxidation but were quantifiable after reaction, these are denoted NA.

ng L-1	Influent 1	Influent 2	Effluent 1	Effluent 2	% change after TOP	Influent 1	Influent 2	Effluent 1	Effluent 2
PFBA	7.2	9.8	7.4	9.7		N.A.	N.A.	N.A.	N.A.
PFPeA	7.4	9.0	4.1	8.8		712	574	295	289
PFHxA	5.8	6.2	5.8	7.5		356	306	176	235
PFHpA	3.9	4.9	2.3	3.6		392	290	207	202
PFOA	5.8	7.6	4.7	5.2		213	236	146	152
PFNA	1.1	1.6	0.8	1.5		311	338	148	156
PFDA	3.7	5.5	2.8	3.8		951	1374	634	785
PFUnDA	0.5	0.7	<MQL	<MQL		N.A.	N.A.	NA	N.A.
PFDoDA	0.2	0.5	<MQL	<MQL		N.A.	N.A.	N.A.	N.A.
PFBS	1.0	1.4	1.0	1.4		147	137	114	149
L-PFHxS	1.7	2.4	2.1	2.3		127	135	146	172
L-PFOS	2.0	3.1	1.9	3.4		147	140	170	166
Br-PFOS	2.1	3.2	1.9	3.3		124	151	158	162

Quality control

Instrumental detection limit of EOF-CIC was determined by the lowest point in calibration curve and using the sample dilution factor. Method detection limits (MDL) were determined using background concentration in procedural blanks and were calculated using average signal

plus three times the standard deviation. MDLs were determined on a batch-to-batch basis and varied between 200-900 ng L^{-1} F.

Method quantification limits (MQLs) of individual analytes were calculated based on 1) the instrument quantification limits (IQLs), 2) instrumental matrix effects (i.e. matrix dependent ion suppression or enhancement) and extraction recoveries and 3) sample concentration factors. Thus, different MQLs for influent and effluent are presented. Instrument quantification limits were based on the lowest point in a calibration curve that maintained at least a signal to noise of 10 and instrumental matrix effects and recoveries were calculated on samples spiked before extraction (n=3), after extraction (n=3) and standard solutions (n=3). MQL may exceed reported concentrations in cases where signal to noise in samples were acceptable (i.e., >10). Thus, MQLs calculated from calibration curves, recovery and matrix effects served as basis for MQL determination but not absolute. However, in cases of blank signals, the average signals in blanks (n=7) plus three times the standard deviation of the blank signal was calculated on an extraction batch basis. If exceeding the value derived from lowest point in a calibration curve, this blank determined MQL was used.

Table S8. Method quantification limits (MQLs) of individual analytes were calculated accounting for 1) the instrument quantification limits (IQLs), 2) instrumental matrix effects (i.e. matrix dependent ion suppression or enhancement) and extraction recoveries and 3) sample concentration factors. Thus, different MQLs for influent and effluent are presented. Instrument quantification limits were based on the lowest point in a calibration curve that maintained at least a signal to noise of 10 and instrumental matrix effects and recoveries were calculated on samples spiked before extraction (n=3), after extraction (n=3) and standard solutions (n=3). In cases of blank signals, the average signals in blanks (n=7) plus three times the standard deviation of the blank signal was calculated. If exceeding the value derived from lowest point in a calibration curve, this blank derived MQL was used.

**Compound name**	**Analytical method**	**MQL effluent (ng/L)**	**MQL influent (ng/L)**
Rosuvastatin	B (ESI-)	4.72	6.89
Citalopram	B (ESI+)	0.43	1.02
Efavirenz	B (ESI-)	1.75	1.76
Ezetimibe	B (ESI-)	0.44	0.50
Atorvastatin	B (ESI-)	20.06	13.41
Ticagrelor	B (ESI+)	4.60	5.67
Sitagliptin	B (ESI+)	0.52	0.73
Bicalutamide	B (ESI-)	0.39	0.42
Fluconazole	B (ESI+)	2.57	4.17
Flecainide	B (ESI-)	1.79	2.13
Fluocloxacillin	B (ESI+)	4.50	14.19
Fluoxetine	B (ESI+)	3.74	7.38
Bendroflumethiazide	B (ESI-)	0.42	0.54
5-fluorouracil	B (ESI-)	NR	NR
8-hydroxy efavirenz	B (ESI-)	2.18	2.18
Desmethyl citalopram	B (ESI+)	0.57	1.75
Celecoxib carboxylic acid	B (ESI-)	1.71	2.34
4-hydroxy atorvastatin	B (ESI-)	36.52	20.44
Deshydroxyethoxy ticagrelor	B (ESI+)	24.52	30.90

Seproxetine	B (ESI+)	37.15	78.76
Enzalutamide carboxylic acid	B (ESI-)	0.26	0.31
Sitagliptin N-sulfate	B (ESI-)	1.33	1.16
Celecoxib	B (ESI+)	4.46	6.78
Ciprofloxacin	B (ESI+)	NR	NR
Emtricitabine	B (ESI+)	0.57	0.91
Rufinamide	B (ESI+)	0.83	1.08
Rufinamide carboxylic acid	B (ESI+)	2.27	4.33
Flecainide Meta-O-dealkylated	B (ESI+)	0.12	0.12
Diflufenican	B (ESI+)	4.32	13.33
Fluroxipyr	B (ESI-)	5.28	6.79
Fipronil	B (ESI-)	0.17	0.20
Fipronil-sulfone	B (ESI-)	0.15	0.18
Benzovindiflupyr	B (ESI+)	0.66	1.24
Fluopyram	B (ESI+)	0.30	0.49
Fluxapyroxad	B (ESI-)	0.03	0.04
Flufenacet	B (ESI+)	0.30	0.49
Fludioxonil	B (ESI-)	3.30	7.73
Bisphenol AF	B (ESI-)	0.33	0.41
TFMS	C (ESI-)	0.87	1.41
PFEtS	C (ESI-)	0.63	1.45
PFPrS	C (ESI-)	0.59	0.68
PFBS	A1 (ESI-)	0.24	0.27
PFPeS	A1 (ESI-)	0.19	0.23
PFHxS	A1 (ESI-)	0.18	0.21
PFHpS	A1 (ESI-)	0.17	0.20
PFOS	A1 (ESI-)	0.23	0.23
PFNS	A1 (ESI-)	0.37	0.46
PFDS	A1 (ESI-)	0.20	0.27
PFUnDS	A1 (ESI-)	0.10	0.15
PFDoDS	A1 (ESI-)	0.20	0.38
PFTrDS	M1 (ESI-)	0.12	0.37
TFA	C (ESI-)	243.18	101.23
PFPrA	C (ESI-)	1.86	3.32
PFBA	A1 (ESI-)	0.99	1.03
PFPeA	A1 (ESI-)	0.43	0.42
PFHxA	A1 (ESI-)	0.28	0.28
PFHpA	A1 (ESI-)	0.06	0.06
PFOA	A1 (ESI-)	0.43	0.43
PFNA	A1 (ESI-)	0.22	0.24
PFDA	A1 (ESI-)	0.14	0.16
PFUnDA	A1 (ESI-)	0.18	0.25
PFDoDA	A1 (ESI-)	0.14	0.25
PFTrDA	A1 (ESI-)	0.14	0.26
PFTDA	A1 (ESI-)	0.22	0.31
PFHxDA	A1 (ESI-)	0.55	1.80

PFOcDA	A1 (ESI-)	0.30	9.35
3:3 FTCA	A1 (ESI-)	1.64	1.73
5:3 FTCA	A1 (ESI-)	0.35	0.34
7:3 FTCA	A1 (ESI-)	0.23	0.27
6:2 FTUCA	A1 (ESI-)	0.21	0.25
8:2 FTUCA	A1 (ESI-)	0.15	0.19
10:2 FTUCA	A1 (ESI-)	0.12	0.19
4:2 FTSA	A1 (ESI-)	0.05	0.06
6:2 FTSA	A1 (ESI-)	0.03	0.03
8:2 FTSA	A1 (ESI-)	0.14	0.14
10:2 FTSA	A1 (ESI-)	0.11	0.11
6:2 diPAP	A2 (ESI-)	0.03	0.03
8:2 diPAP	A2 (ESI-)	0.06	0.08
6:2/8:2 diPAP	A2 (ESI-)	0.01	0.01
SAmPAP	A2 (ESI-)	3.00	5.45
diSAmPAP	A2 (ESI-)	0.10	0.17
PFHxPA	A1 (ESI-)	1.20	1.43
PFOPA	A1 (ESI-)	1.16	1.98
C6/C6 PFPiA	A1 (ESI-)	0.16	0.22
C6/C8 PFPiA	A1 (ESI-)	0.22	0.31
C8/C8 PFPiA	A1 (ESI-)	0.40	0.66
FBSA	A1 (ESI-)	0.20	0.20
FHxSA	A1 (ESI-)	0.25	0.32
FOSA	A1 (ESI-)	1.19	1.19
FOSAA	A1 (ESI-)	0.01	0.01
MeFOSAA	A2 (ESI-)	0.51	0.51
EtFOSAA	A2 (ESI-)	0.03	0.04
MeFBSA	A1 (ESI-)	28.69	10.97
MeFHxSA	A1 (ESI-)	21.96	19.22
MeFOSA	A1 (ESI-)	13.92	24.59
EtFOSA	A1 (ESI-)	15.07	27.94
ADONA	A1 (ESI-)	0.02	0.02
HFPO-DA	A1 (ESI-)	1.29	1.79
3.6-OPFHpA	A1 (ESI-)	0.04	0.04
PF4OPeA	A1 (ESI-)	0.78	1.10
PFEESA	A1 (ESI-)	0.10	0.11
6:2 Cl-PFESA	A1 (ESI-)	0.03	0.03
8:2 Cl-PFESA	A1 (ESI-)	0.04	0.05
FMeSI	A1 (ESI-)	0.03	0.03
FEtSI	A1 (ESI-)	0.02	0.03
FBSI	A1 (ESI-)	0.05	0.06
PFECHS	A1 (ESI-)	0.03	0.04
5:3 FTB	B (ESI+)	0.65	2.34
AP-FHxSA	B (ESI+)	0.24	0.38
TAmP-FHxSA	B (ESI+)	0.26	0.45
6:2 FTAB	B (ESI+)	2.15	2.89

FSI	C (ESI-)	15.54	19.25
Hexafluorophosphate	C (ESI-)	42.10	49.37
Tetrafluoroborate	C (ESI-)	151.26	110.69

NR. Not reported.

Influent and effluent concentrations

Concentrations of target analytes in each sample are presented in table S10 below. Influent and effluent samples were extracted in triplicates. Reported concentrations herein are the calculated average of triplicates except for sample S2 that refers to a duplicate as one replicate thereof could not be analyzed. The linear isomer of PFOS is reported separately from the sum of branched isomers. For all other PFAS, reported concentration refers to the linear isomer only.

Table S9. Concentration of target analytes. Compounds detected via LC-HRMS screening before a targeted method was developed are indicated in the table. Note: sample S2 refers to the average of a duplicates, while all other samples are averages of triplicates.

ng/L	Identified via LC-HRMS screening?	Influent S1	Influent S2	Influent S3	Effluent S1	Effluent S2	Effluent S3
Rosuvastatin	Yes	431.37	452.47	495.11	517.75	242.89	475.26
Citalopram	Yes	339.78	296.59	102.51	207.32	182.45	138.14
Efavirenz	Yes	5.89	5.49	3.36	2.65	2.80	2.85
Ezetimibe	Yes	61.04	79.33	75.84	32.37	34.29	29.90
Atorvastatin	Yes	ND	ND	38.64	ND	ND	<MQL
Ticagrelor	Yes	7.83	10.85	9.78	<MQL	5.40	<MQL
Sitagliptin	Yes	2238.06	2228.28	1442.07	1258.65	1132.23	781.40
Bicalutamide	Yes	234.82	407.75	216.02	336.95	372.57	207.07
Fluconazole	Yes	129.89	144.12	124.87	101.41	106.25	68.18
Flecainide	Yes	35.18	42.27	32.05	45.79	46.91	27.48
Flucloxacillin	No	16.05	<MQL	38.03	59.86	41.47	9.23
Fluoxetine	No	21.02	53.56	<MQL	5.70	15.04	10.87
Bendroflumethiazide	Yes	<MQL	<MQL	<MQL	<MQL	<MQL	0.85
5-fluorouracil	No	NA	NA	NA	NA	NA	NA
8-hydroxy efavirenz	Yes	61.33	80.62	19.10	4.25	<MQL	3.44
Desmethyl citalopram	Yes	255.18	232.50	52.87	226.16	174.55	108.94
Celecoxib carboxylic acid	Yes	239.13	335.84	271.41	406.53	505.82	349.72
4-hydroxy atorvastatin	Yes	ND	ND	100.03	ND	ND	<MQL
Deshydroxyethoxy ticagrelor	Yes	72.01	51.84	38.03	<MQL	<MQL	<MQL
Seproxetine	No	ND	ND	ND	ND	ND	ND
Enzalutamide carboxylic acid	Yes	28.56	45.28	23.12	42.38	53.77	22.56
Sitagliptin N-sulfate	Yes	44.64	48.36	30.42	52.04	46.34	26.85
Celecoxib	Yes	50.78	21.54	70.14	37.67	42.74	47.03
Ciprofloxacin	No	NA	NA	NA	NA	NA	NA
Emtricitabine	No	362.51	395.36	431.01	37.59	45.48	38.25
Rufinamide	No	6.06	4.82	9.63	0.88	<MQL	0.97
Rufinamide carboxylic acid	Yes	36.23	57.00	41.13	28.12	49.48	36.85
Flecainide Meta-O-dealkylated	Yes	45.10	61.14	73.79	18.81	7.95	17.05

Diflufenican	No	ND	ND	ND	ND	ND	ND
Fluroxipyr	No	ND	ND	ND	ND	ND	ND
Fipronil	Yes	5.43	7.67	2.45	6.19	7.95	2.78
Fipronil-sulfone	Yes	0.56	1.07	0.66	0.77	0.88	0.85
Benzovindiflupyr	No	ND	ND	ND	ND	ND	ND
Fluopyram	No	4.90	1.12	4.26	0.91	2.07	0.87
Fluxapyroxad	No	0.26	0.66	0.18	0.22	0.57	0.29
Flufenacet	No	ND	ND	ND	ND	ND	ND
Fludioxonil	Yes	8.72	<MQL	<MQL	<MQL	<MQL	3.85
Bisphenol AF	No	ND	ND	ND	ND	ND	ND
TFMS	No	2.75	4.60	3.03	3.55	4.32	4.75
PFEtS	No	ND	ND	ND	ND	ND	ND
PFPrS	No	ND	ND	ND	ND	ND	ND
PFBS	No	0.71	1.02	0.65	0.90	0.91	0.42
PFPeS	No	ND	ND	ND	<MQL	<MQL	<MQL
PFHxS	No	1.35	1.80	1.19	1.43	1.34	0.84
PFHpS	No	ND	ND	ND	ND	ND	ND
L-PFOS	No	1.37	2.20	1.19	1.11	2.04	0.95
Br-PFOS	No	1.11	2.06	0.68	1.51	1.73	0.69
PFNS	No	ND	ND	ND	ND	ND	ND
PFDS	No	ND	ND	ND	ND	ND	ND
PFUnDS	No	ND	ND	ND	ND	ND	ND
PFDoDS	No	ND	ND	ND	ND	ND	ND
PFTrDS	No	ND	ND	ND	ND	ND	ND
TFA	No	322.40	745.90	281.72	153.27	132.13	128.18
PFPrA	No	7.79	8.13	4.62	4.50	7.35	5.85
PFBA	No	<MQL	<MQL	<MQL	<MQL	<MQL	<MQL
PFPeA	No	1.04	1.56	<MQL	1.37	3.03	1.16
PFHxA	No	1.62	2.04	1.48	3.31	3.20	1.70
PFHpA	No	1.00	1.68	1.00	1.11	1.77	0.97
PFOA	No	2.73	3.24	1.65	3.22	3.42	1.58
PFNA	No	0.34	0.49	0.37	0.53	0.94	0.42
PFDA	No	0.39	0.40	0.31	0.43	0.48	0.27
PFUnDA	No	ND	ND	ND	ND	ND	ND
PFDoDA	No	ND	ND	ND	ND	ND	ND
PFTrDA	No	ND	ND	ND	ND	ND	ND
PFTDA	No	ND	ND	ND	ND	ND	ND
PFHxDA	No	ND	ND	ND	ND	ND	ND
PFOcDA	No	ND	ND	ND	ND	ND	ND
3:3 FTCA	No	ND	ND	ND	ND	ND	ND
5:3 FTCA	No	0.48	ND	0.73	0.49	ND	<MQL
7:3 FTCA	No	ND	ND	ND	ND	ND	ND
6:2 FTUCA	No	ND	ND	<MQL	0.22	ND	ND
8:2 FTUCA	No	ND	ND	ND	ND	ND	ND
10:2 FTUCA	No	ND	ND	ND	ND	ND	ND
4:2 FTSA	No	ND	ND	ND	ND	ND	ND

6:2 FTSA	No	0.83	<MQL	<MQL	0.89	<MQL	<MQL
8:2 FTSA	No	1.87	6.24	5.93	1.72	1.07	0.92
10:2 FTSA	No	0.15	0.62	0.67	ND	ND	ND
6:2 diPAP	No	0.28	0.48	0.05	ND	ND	<MQL
8:2 diPAP	No	0.09	<MQL	ND	ND	ND	ND
6:2/8:2 diPAP	No	0.06	0.14	ND	ND	ND	ND
SAmPAP	No	ND	ND	ND	ND	ND	ND
diSAmPAP	No	ND	ND	ND	ND	ND	ND
PFHxPA	No	ND	ND	ND	ND	ND	ND
PFOPA	No	ND	ND	ND	ND	ND	ND
C6/C6 PFPiA	No	ND	ND	ND	ND	ND	ND
C6/C8 PFPiA	No	ND	ND	ND	ND	ND	ND
C8/C8 PFPiA	No	ND	ND	ND	ND	ND	ND
FBSA	No	0.30	<MQL	0.26	0.30	0.33	0.52
FHxSA	No	<MQL	<MQL	ND	<MQL	<MQL	ND
FOSA	No	<MQL	<MQL	<MQL	<MQL	<MQL	<MQL
FOSAA	No	ND	ND	ND	ND	ND	ND
MeFOSAA	No	ND	ND	ND	ND	ND	ND
EtFOSAA	No	<MQL	0.25	0.14	<MQL	0.12	0.17
MeFBSA	No	ND	ND	ND	ND	ND	ND
MeFHxSA	No	ND	ND	ND	ND	ND	ND
MeFOSA	No	ND	ND	ND	ND	ND	ND
EtFOSA	No	ND	ND	ND	ND	ND	ND
ADONA	No	ND	ND	ND	ND	ND	ND
HFPO-DA	No	ND	ND	ND	ND	ND	ND
3,6-OPFHpA	No	ND	ND	ND	ND	ND	ND
PF4OPeA	No	ND	ND	ND	ND	ND	ND
PFEESA	No	ND	ND	ND	ND	ND	ND
6:2 Cl-PFESA	No	ND	ND	ND	ND	ND	ND
8:2 Cl-PFESA	No	ND	ND	ND	ND	ND	ND
FMeSI	No	0.17	1.02	0.29	0.24	0.52	0.29
FEtSI	No	ND	ND	ND	ND	ND	ND
FBSI	No	ND	ND	ND	ND	ND	ND
PFECHS	No	ND	ND	ND	ND	ND	ND
5:3 FTB	No	ND	ND	ND	ND	ND	ND
AP-FHxSA	No	ND	ND	ND	ND	ND	ND
TAmP-FHxSA	No	ND	ND	ND	ND	ND	ND
6:2 FTAB	No	ND	ND	ND	ND	ND	ND
FSI	No	ND	ND	ND	ND	ND	ND
Hexafluorophosphate	No	188.02	133.50	62.67	172.49	80.06	70.20
Tetrafluoroborate	No	269.92	97.93	367.87	55.17	19.74	65.81
EOF		1781	3305	1259	1767	2341	1382

Sitagliptin predicted influent concentrations

Data was retrieved from the Swedish National Board of Health and Welfare [7], which indicated the number of patients prescribed sitagliptin, in the county where the treatment plant of this study was located. Approximately half of these were assumed to be connected to the treatment plant based on person equivalents of the treatment plants and number of persons living in the county. Assuming a daily dose of 100 mg [8] and a mass flow of 40000 m3/24h, the influent ∑concentration of sitagliptin (including potential transformation products) was 3100 ng L^{-1}. Accounting for the percentage of sitagliptin excreted unchanged (80%), the concentration of sitagliptin parent was predicted to approximately 2500 ng L^{-1}. The number of patients prescribed sitagliptin was assumed stable throughout the year, as the total number of patients between January and December of 2023 differed by <5%.

LC-HRMS screening

Screening workflow

Suspect lists of fluorinated pharmaceuticals and related compounds were compiled from Inoue et al. 2020 [9] that cataloged 340 fluorinated pharmaceuticals approved by the United States Food and Drug Administration between 1954 and 2019. Because numerous of the listed pharmaceuticals in Inoue et al., 2020, are either not approved or currently prescribed in Sweden, the list was curated further with data from the Swedish National Board of Health and Welfare [10] to compile a relevant list of ~100 fluorinated pharmaceuticals sold under prescription in Sweden as of 2023. A pharmaceutical was considered irrelevant if they were not prescribed in previous years or had few prescriptions (e.g., <10). From these compounds, known metabolites and transformation products were composed from literature and drugbank.com. Fluorinated veterinary pharmaceuticals approved in Sweden were compiled from the Swedish Medical Product Agency [11] online database. Further, PFAS suspect lists (PMT PFAS [12]; aqueous film-forming foam [AFFF] related [13]) and lists of fluorinated pesticides, relevant for Sweden, were retrieved from literature [14]. Moreover, fluorinated illicit drugs curated and screened for. Suspect list curated within the scope of the present study (i.e., lists of pharmaceuticals, metabolites veterinary pharmaceuticals and illicit drugs) can be found in SI excel file. The rest of the suspect lists are referred to respective references. For data processing, adducts of [M-H]- and [M+H]+, [M+NH4]+, [M+Na]+, were checked in negative and positive ionization, respectively. The HRMS instrument operated in a data-independent acquisition mode (MS^e). While spectral deconvolution occurs in post processing of the UNIFY software, this acquisition mode generally leads to a noisier MS2 spectra than e.g., a data-dependent acquisition mode. Manually inspecting fragments in MS2 by isolating a fragment m/z and confirming its co-elution with corresponding precursor therefore aided in the confidence of reported fragment data.

Standards were purchased of 24 tentatively identified compounds that were later confirmed. This included atorvastatin, bendroflumethiazide, efavirenz, ticagrelor, celecoxib, flecainide, ezetimibe, citalopram, fluconazole, bicalutamide, rosuvastatin, sitagliptin, atorvastatin 4-hydroxy, efavirenz 8-hydroxy, enzalutamide carboxylic acid, sitagliptin N-sulfate, rufinamide carboxylic acid, ticagrelor deshydroxyethoxy, citalopram desmethyl, celecoxib carboxylic acid, fipronil, fipronil sulfone and fludioxonil. In addition, mass labelled standards of citalopram, rosuvastatin and fluoxetine were also acquired to use in the quantification workflow following LC-HRMS screening.

Tentatively identified compounds
All reported tentatively identified compounds below had <5 ppm mass error from theoretical monoisotopic mass of corresponding $[M-H]^-$ and an isotopic ratio match within 30 RMS % (UNIFY 1.9.4) together with either library MS2 match or diagnostic MS2 fragments. A summary of tentative structures, mass errors, RTs, isotopic fit and fragments are provided in table S11.

N-methylperfluorobutane sulfonamidoacetic acid (MeFBSAA) was detected at a level 2b confidence level, based on fragments m/z 311.9757, 282.9473 and 218.9866 matching previously reported MS2 fragments confirmed with standard [15]. Considering perfluoroalkane sulfonamidoacetic acids have been considered as intermediate environmental transformation products [16] and MeFBSAA has specifically been reported as an anaerobic biotransformation product of N-methyl perfluorobutanesulfonamido ethanol [17]. MeFBSAA was therefore hypothesized to be the result of degradation of an unknown precursor compound during the wastewater treatment processes.

Hydrogen-substituted 1:2 perfluoroether sulfonic acid (1:2 H-PFESA) was assigned to m/z 246.9503, RT 2.65 minutes (corresponding to the exact mass of $[C_3F_6HSO_4]^-$ at -0.6 ppm mass error) and was detected in both influent and effluent. Previously, this compound was reported with four diagnostic fragments, making it possible to assign a probable structure at a confidence level of 2. In the present study, three of these fragments were present while the fourth diagnostic fragment (m/z 68.9957 corresponding to $[CF_3]^-$) was missing, leading to some ambiguity of the exact position of the substituted hydrogen. However, the loss of HF (fragment m/z 226.9433) indicates the hydrogen positioned on the alpha carbon, and together with two other diagnostic fragments previously reported in literature (m/z 146.9859, 166.9925), this feature was assigned to 1:2 H-PFESA at a level 2(b) confidence level.

Bicalutamide is a racemate and is known to undergo stereoselective metabolism where (S)-bicalutamide is metabolized directly via glucuronidation, while the main metabolic pathway for (R)-bicalutamide is hydroxylation followed by glucuronidation [18]. However, no glucuronide conjugated bicalutamide or derivate was detected in the screening. As no specific precaution was taken to prevent deconjugation in the wastewater samples, cleavage of the glucuronide side chain during sample storage could have occurred [19]. In total, three compounds suspected to be hydroxy-bicalutamide metabolites and derivates were tentatively identified herein. In two of them, TP-425A and TP-425B, there is some ambiguity regarding sulfation on the hydroxyl group. This is discussed in more detail in the following section.

Bicalutamide TP-446 (m/z 445.0475, RT 6.58, annotated as $[C_{18}H_{13}F_4N_2O_5S]^-$ at -2.6 ppm mass error) was assigned to a hydroxy-bicalutamide with an undefined point of hydroxylation, based on the formation of the same characteristic fragment ions as bicalutamide (parent) at m/z 185.0316 ($[C_8H_4F_3N_2]^-$) and 255.0376 ($[C_{11}H_6F_3N_2O_2]^-$). TP-525A (m/z 525.0049, RT 5.01, corresponding to $[C_{18}H_{13}F_4N_2O_8S_2]^-$ at -1.1 ppm mass error) was assigned to a sulfated hydroxy-bicalutamide with the O-SO_3- moiety present on the fluorophenyl ring. This is indicated in the MS2 data, with m/z 173.9781 and 189.0018, corresponding to sulfonyl- and methylsulfonyl hydroxy-fluorophenyl fragments, present together with the characteristic bicalutamide fragments at m/z 185.0331 and 255.0387. Co-eluting with m/z 525.0049 in MS1, m/z 445.0490 (corresponding to deprotonated hydroxy-bicalutamide) was present at a much higher detector response (>10 times), possibly due to significant in-source fragmentation in MS1 leading to loss of the $[SO_3]^-$ group. The shorter retention time of TP525A compared to TP-446 provides support for the presence of a SO_3- group, increasing its polarity and decreasing its RT. As a

likely consequence of the significant in-source fragmentation in MS1, there is an absence of a signal corresponding to an (H)SO_3- or (H)SO_4- fragment ion in MS2. Consequently, it cannot be unambiguously excluded that m/z 525.0049 peak could be an instrument artefact due to a sulfur trioxide adduct formation in the ion source. TP-525B (m/z 525.0051, RT 5.58, [$C_{18}H_{13}F_4N_2O_8S_2$]- at -0.3 ppm mass error) was assigned to another sulfated hydroxy-bicalutamide. MS2 fragments (m/z 255.0376 and 271.0325) indicate the O-SO_3- moiety was present at the methylpropane-amide. MS2 spectrum of TP-525B contained m/z 96.9592, corresponding to $[HSO_3]^-$ at a slightly elevated (10 ppm) mass error. TP-525B did not show an in-source fragment ion corresponding to hydroxy-bicalutamide in MS1; however, a fragment ion corresponding to hydroxy-bicalutamide was present in MS2. The reason for this discrepancy of TP-525A and TP-525B tendencies to undergo in-source fragmentation may be due to TP-525A having a more exposed location of O-SO_3- group, leading to more favorable cleavage of the S-O bond in the ion source.

Two features suspected to be metabolites or transformation products of sitagliptin were further identified; one of them (sitagliptin N-sulfate) was confirmed with standard. Another feature, at m/z 448.1204 was annotated as $[C_{18}H_{16}F_6N_5O_2]^-$ and was assigned to N-acetyl sitagliptin/sitagliptin TP449 [20] at CL3. A library match of fragment m/z 191.0543 (3.8 ppm mass error) corresponding to the trifluoromethyl-triazolopiperazine side chain was present, which is present in a library spectra [21]. Furthermore, a diagnostic in-silico fragment of m/z 270.0064, corresponding to [$C_{12}H_9F_3N_2O_2$]- was present.

Semi quantification of tentative detected compounds was performed using 1-point external calibration. MeFOSAA was used for MeFBSAA; PFBS for 1:2 H-PFESA; bicalutamide was used for its TPs and sitagliptin for TP-449.

Table S10. List of tentatively identified compounds.

Abbreviation	Chemical formula (neutral)	Tentative structure	Confidence level	Observed m/z	RT	Mass error (ppm)	UNIFY Isotope RMS%	Fragment 1 (m/z, formula)	Fragment 2 (m/z, formula)	Fragment 3 (m/z, formula)	Fragment 4 (m/z, formula)
**MeFBSAA**	C7H6F9NO4S	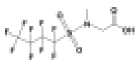	CL2(b)	369.9792	6.46	2.6	25	311.97567 (C5H3F9NO2S−)	282.94733 (C4F9SO2−)	218.98659 (C4F9−)	
**1:2 H-PFESA**	C3F6H2SO4	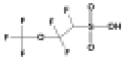	CL2(b)	246.9503	2.65	-0.6	25	146.98591 (C3F5O-)	166.99253 (C3HF6O-)	226.94325 (C3F5O4S-)	
**Bicalutamide TP-446**	C18H14F4N2O5S2	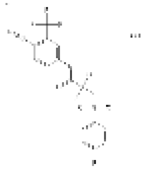	CL3	445.0475	6.58	-2.6	22	185.0316 (C8H4F3N2-)	255.0376 (C11H6F3N2O2-)		
**Bicalutamide TP-526A**	C18H14F4N2O8S2	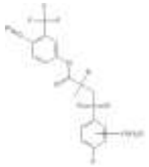	CL2(b)	525.0049	5.01	-1.1	15	185.0331 (C8H4F3N2-)	255.0387 (C11H6F3N2O2-)	173.9781 (C6H3FO3S-)	189.0018 (C7H6FO3S-)
**Bicalutamide TP-526B**	C18H14F4N2O8S2	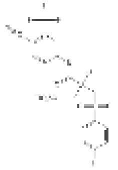	CL3	525.0051	5.58	-0.3	28	271.0325 (C11H6F3N2O3)	255.0376 (C11H6F3N2O2-)	96.9592 (HSO4-)	
**Sitagliptin TP449**	C18H17F6N5O2	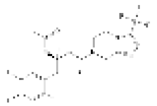	CL3	448.1204	5.65	-2.1	11	191.0543 (C6H6F3N4-)	270.0640 (C12H9F3N2O2-)		

Method performance testing

Quantification of fluorinated pharmaceuticals, metabolites and pesticides

The following section refers to LC-MS/MS methods used for quantification of low fluorinated compounds unless otherwise stated. Method performance of conventional PFAS analysis in our lab is referred to previous publication [22]. Linearity (minimum R^2 coefficient >0.99) of the methods were evaluated based on target compounds in solvent (i.e., 1:1 ammonium acetate: methanol) using 10 calibration points, with most compounds achieving an R^2 coefficient >0.99 of the analyte response normalized to internal standard in the range of 0.1-100 ng mL^{-1} with seven calibration points.

For quantification of pharmaceuticals, pesticides and related compounds were performed using calibration curves of 5-10 points with the response of native compounds normalized to the response of the internal standards. In ESI-, d3-rosuvastatin was used as internal standard, and in ESI+, d3-rosuvastatin, d6-citalopram, or a combined response of both, was used. For quantification of fluoxetine, d6-fluoxetine was used. The selection of internal standards in ESI+ was derived from experimentally determined measurement accuracy, where the internal standard(s) with the demonstrated best fit were selected. Due to the difference in matrix effect in influent and effluent samples, internal standard selection therefore differed, in some cases, between influent and effluent.

The accuracy of the quantification method was determined via matrix-matched calibration curves obtained via extracted influent and effluent wastewater. Matrix extracts were added to a calibration curve of 10 points and treated the same way as samples, i.e., equal concentration of matrix as real samples. Because the matrix already contained analytes, the accuracy was determined on two or three calibrations points above the native concentration in the samples.

Table S11. Accuracy of the quantification in influent and effluent wastewater of low-fluorinated compounds

	Influent		Effluent	
	Accuracy (%)	Stdev	Accuracy (%)	Stdev
Fludioxonil	584	73.1	221	61.0
Efavirenz	264	16.3	213	4.3
Efavirenz 8-hydroxy	260	25.8	204	49.7
Bisphenol AF	90	10.8	116	6.6
Fluoxapyroxad	106	7.5	125	5.5
Ezetimibe	86	5.0	98	0.9
Celecoxib carboxy	96	3.3	94	4.1
Flecainide	98	6.6	94	2.3
Bendroflumethiazide	58	7.0	71	12.3
Bicalutamide	108	12.3	110	11.2
Fipronil	94	11.7	136	3.2
Fipronil sulfone	98	13.9	135	5.6
Enzalutamide carboxy	69	2.9	66	4.4
Rosuvastatin	116	21.6	109	13.9
Sitagliptin N-sulfate	292	6.5	225	24.1
Atorvastatin	126	6.6	84	4.0
Atorvastatin 4-hydroxy	93	5.5	70	3.1
Rufinamide	110.2	7.2	89.2	10.4

Rufinamide acid	86.6	10.1	75.6	1.8
Emtricitabine	112.0	2.9	78.0	1.2
Seproxetine	35.2	1.0	35.5	2.1
Fluconazole	144.4	3.9	118.8	3.6
Fluoxetine	68.9	3.7	72.1	4.8
Citalopram desmethyl	64.4	1.4	72.1	7.2
Citalopram	129.4	4.1	110.6	2.3
Ciprofloxacin	87.6	8.3	95.5	4.0
Flecainide dealkylated	122.6	14.6	129.4	5.9
Flufenacet	92.6	5.6	87.4	3.5
Celecoxib	132.0	13.5	121.9	7.9
Diflufenican	110.9	9.8	64.9	15.0
Fluopyram	92.6	16.3	87.4	12.0
Benzovindiflupyr	147.7	3.9	136.5	4.4
Sitagliptin*	97.2	4.7	89.6	4.5
Flucloxacillin	23.8	2.5	42.8	1.8
Ticagrelor deshydroxyethoxy**	126.9	20.8	63.2	6.7
Ticagrelor**	111.2	3.3	78.0	2.5

* stdev calculated on two concentration points (influent and effluent)

** stdev calculated on two concentration points (influent)

Accuracy of the quantification was determined in wastewater effluent and influent matrix at two or three concentrations levels. For most compounds in ESI-, the accuracy was 92% (SD 22%) and 108% (SD 24%) for wastewater effluent and influent, respectively. For ESI+ the corresponding accuracy was 101% (SD 23%) and 95% (SD 17%), respectively. However, the accuracy of efavirenz, 8-hydroxy efavirenz, sitagliptin N-sulfate and fludioxonil was inadequate; concentrations of these compounds are therefore reported on an order of magnitude basis.

Inter-day repeatability of LC-MS/MS determinations, expressed as CV of measured concentrations in QC samples (n=4), was between 3-32% (median 15%) for all 32 compounds, including conventional PFAS, quantitatively reported. For compounds with higher CV, concentration was reported in the order of magnitude range (n=8).

Evaluation of multi-sorbent solid phase extraction for EOF analysis

The performance of the applied multi-sorbent-SPE method was evaluated and compared to previously published single-sorbent-SPE protocols [2, 23]. This included 1) extraction of all samples with both methods to compare EOF concentrations and 2) a spike recovery test to evaluate extraction recovery efficiency of the two methods. The recovery of individual compounds was assessed in both influent and effluent wastewater where samples were spiked before (n=3) and after extraction (n=3) and non-spiked samples (n=3). Samples were fortified with a total of 114 fluorinated compounds. Three synthetic inorganic compounds containing fluorine were included to evaluate co-extraction of inorganic species, an effect seen in other EOF methods using weak anion exchange SPE [24]. The recovery (table S13) was calculated by subtracting peak areas of non-spiked samples from samples spiked before extraction and dividing them with matrix-subtracted peak areas spiked after extraction. For assessing matrix effects (table S14), peak areas of non-spiked samples were subtracted from samples spiked after extraction and divided by analyte peak area of a standard solution without matrix. The spike

concentration aimed to reflect approximate matrix concentrations and was 25 ng/L for conventional PFAS and pesticides and 800 ng/L for pharmaceuticals and inorganic substances. The suit of compounds was a mix of conventional highly fluorinated perfluorinated acids and precursors (n=73) and weakly fluorinated (e.g., pharmaceutical or pesticide related compounds) (n=38) substances and inorganic substances (n=3). While a large proportion of the conventional PFAS were structurally similar (e.g., containing a fluorocarbon chain with an acidic headgroup), the inclusion of a wide range of pharmaceutical- and pesticide-related compounds enabled evaluation of the method extraction recovery in an extended chemical space.

Table S12. Extraction recoveries of multi-sorbent SPE and WAX-SPE in influent and effluent wastewater. Average and standard deviation is presented and is based on triplicate samples.

	msSPE-effluent		WAX-SPE-effluent		msSPE-influent		WAX-SPE-influent	
	Recovery (%)	SD	Recovery (%)	SD	Recovery (%)	SD	Recovery (%)	SD
Rosuvastatin	88.5	4.3	97.9	10.2	65.9	9.5	88.5	12.3
Citalopram	94.0	0.8	62.0	7.5	100.1	1.8	50.1	10.4
Efavirenz	92.0	3.5	96.6	7.0	84.8	9.1	84.0	11.5
Ezetimibe	95.9	3.5	97.1	4.3	86.6	10.7	79.1	11.3
Atorvastatin	27.5	2.3	64.7	8.6	40.2	10.2	30.5	12.8
Ticagrelor	85.0	1.2	91.4	5.8	79.9	8.4	82.4	6.3
Sitagliptin	61.3	4.4	0.8	0.2	87.4	1.9	0.4	0.1
Bicalutamide	98.9	3.5	97.4	6.0	89.9	9.0	88.9	11.1
Fluconazole	101.9	0.8	73.3	9.1	97.7	5.5	64.0	4.4
Flecainide	99.4	5.0	24.8	4.5	92.0	8.3	12.6	2.5
Flucloxacillin	79.4	0.9	74.6	11.8	75.5	18.9	68.7	8.1
Fluoxetine	89.6	4.8	88.6	4.6	108.7	7.6	70.0	11.8
Bendroflumethiazide	93.3	3.0	96.9	7.6	83.8	5.8	81.2	7.4
5-fluorouracil	<1%	NA	<1%	NA	<1%	NA	<1%	NA
Efavirenz 8-hydroxy	64.9	3.3	77.3	2.4	57.7	5.6	64.9	12.6
Citalopram desmethyl	89.4	2.2	65.2	5.4	93.1	8.1	53.0	11.2
Celecoxib carboxylic acid	100.8	3.7	105.3	7.6	92.2	10.3	85.3	10.3
Atorvastatin 4-hydroxy	28.8	2.4	86.3	13.4	61.5	14.4	35.8	14.2
Ticagrelor deshydroxyethoxy	88.0	1.4	91.2	7.2	80.7	7.3	79.4	8.0
Seproxetine	84.6	4.7	70.3	2.1	92.6	17.3	47.1	20.4
Enzalutamide carboxylic acid	93.6	2.7	95.1	5.6	89.0	8.6	85.8	8.7
Sitagliptin N-sulfate	91.5	7.0	96.7	8.2	88.9	10.1	83.2	11.7
Celecoxib	100.0	2.0	98.5	6.0	95.0	3.2	83.2	21.2
Ciprofloxacin	<1%	NA	<1%	NA	<1%	NA	<1%	NA
Emtricitabine	92.0	3.6	0.4	0.0	77.9	9.8	0.4	0.1
Rufinamide	98.8	1.9	98.2	1.7	90.7	8.9	78.4	3.8
Rufinamide acid	97.3	3.4	97.7	6.6	90.8	7.6	78.6	6.4
Flecainide meta-o-dealkylated	98.0	2.3	0.9	0.1	91.0	7.7	0.5	0.0
Diflufenican	93.7	19.0	76.5	2.5	61.2	5.0	59.5	7.2
Fluroxipyr	119.6	24.9	85.4	7.1	104.6	18.4	91.1	0.8
Fipronil	94.6	2.8	105.3	9.0	84.5	7.5	92.6	10.2
Fipronil sulfone	101.6	3.2	102.7	7.7	88.7	8.6	90.1	11.1
Benzovindiflupyr	98.8	1.5	97.0	2.9	86.3	2.9	87.4	0.5
Fluopyram	102.5	2.5	97.5	3.0	105.0	6.4	103.0	7.6
Fluoxapyroxad	106.2	2.8	101.2	6.5	92.7	6.3	92.9	14.9
Flufenacet	100.1	2.1	97.1	4.4	104.1	5.0	113.4	18.3
Fludioxonil	88.8	6.1	99.5	31.8	63.3	16.3	87.4	38.8
Bisphenol AF	96.4	4.1	94.9	11.2	88.5	7.7	89.7	2.8
TFMS	78.5	1.2	63.8	18.2	44.8	5.4	57.8	11.1
PFEtS	106.6	13.0	92.8	13.4	70.7	6.0	70.4	26.6
PFPrS	110.7	4.7	102.8	1.4	85.3	6.9	90.3	17.4
PFBS	96.5	2.8	99.8	3.6	92.5	11.4	86.9	8.8
PFPeS	97.6	2.3	102.6	4.8	90.5	10.4	84.0	7.6
PFHxS	97.0	3.3	100.7	4.0	94.7	12.2	85.3	9.3
PFHpS	98.0	3.3	102.1	5.4	93.2	9.9	84.6	8.2

PFOS	95.6	4.5	103.8	2.5	88.0	11.2	88.8	5.4
PFNS	96.8	4.4	96.0	4.7	83.5	15.8	78.7	3.2
PFDS	91.0	5.7	85.8	5.0	77.7	15.5	77.1	4.3
PFUnDS	88.0	4.8	86.7	6.6	77.2	9.7	75.9	5.5
PFDoDS	90.2	6.3	79.7	6.1	75.7	0.7	65.1	3.2
PFTrDS	98.6	10.8	83.0	4.9	72.4	8.4	64.7	4.0
TFA	9.6	2.9	5.4	0.6	21.9	4.4	22.8	2.3
PFPrA	62.6	7.6	45.9	9.2	37.3	3.7	61.2	7.4
PFBA	127.9	6.3	101.4	2.1	72.6	13.7	61.8	4.2
PFPeA	97.1	2.7	103.9	2.7	89.4	8.0	82.6	2.6
PFHxA	93.5	2.6	101.9	3.0	89.5	10.4	82.3	6.4
PFHpA	94.8	3.0	103.0	3.7	87.4	7.8	84.0	2.9
PFOA	96.4	2.4	99.0	5.6	92.7	10.0	85.1	8.2
PFNA	99.9	5.3	105.1	3.6	93.7	11.1	90.1	7.0
PFDA	98.8	3.9	98.6	5.2	87.7	12.9	83.8	4.6
PFUnDA	95.2	5.4	93.5	3.9	81.6	11.3	76.9	3.5
PFDoDA	89.3	6.3	86.2	5.1	78.1	9.5	74.1	4.6
PFTrDA	89.1	7.0	84.3	6.6	79.5	1.8	75.8	5.4
PFTDA	100.2	8.8	78.0	7.8	78.1	2.7	72.3	5.6
PFHxDA	101.7	17.1	95.2	12.6	71.1	16.7	58.3	2.9
PFOcDA	83.4	4.0	75.0	6.5	60.5	6.1	37.2	3.4
3_3_FTCA	85.3	2.5	93.4	4.4	76.7	9.9	60.5	6.2
5_3 FTCA	70.1	3.7	94.4	3.5	77.0	9.7	59.1	3.6
7_3FTCA	73.1	3.8	78.4	3.7	66.6	16.3	54.3	2.0
6_2 FTUCA	93.9	2.8	90.4	4.2	86.4	9.2	74.6	2.7
8_2_FTUCA	90.4	4.3	88.1	3.4	79.7	14.2	67.8	4.1
10_2_FTUCA	81.7	3.3	78.7	4.0	71.7	13.7	68.1	5.5
4:2 FTSA	100.7	2.8	99.1	5.1	94.1	6.5	84.4	3.8
6:2 FTSA	96.1	5.7	104.7	8.1	95.3	12.4	86.6	8.3
8:2 FTSA	101.2	7.0	102.7	4.7	91.6	11.2	91.1	11.9
10:2 FTSA	93.3	10.8	91.1	8.1	83.2	13.1	83.8	10.2
6:2 diPAP	101.3	5.9	88.6	7.8	75.6	11.1	70.3	5.7
8:2 diPAP	97.1	3.7	69.6	3.5	62.1	10.5	35.0	4.4
6:2-8:2 diPAP	90.7	1.0	84.5	4.2	72.3	11.6	50.8	4.0
SAmPAP	112.8	12.1	79.7	4.5	76.9	6.0	66.5	5.5
diSAmPAP	87.1	2.0	71.4	4.4	60.1	10.0	32.4	2.7
PFHxPA	85.8	10.8	90.6	5.7	89.1	11.5	77.0	2.5
PFOPA	98.2	8.6	85.6	7.9	69.1	9.6	66.0	7.5
6:6 PFPiA	94.2	6.7	89.6	8.1	71.4	18.4	75.7	6.0
6:8 PFPiA	101.2	8.3	91.3	6.1	74.8	12.1	80.6	4.9
8:8 PFPiA	110.6	7.6	97.7	3.5	84.6	6.4	74.5	4.5
FBSA	95.6	3.9	99.7	4.3	87.5	10.7	79.4	8.0
FHxSA	94.4	2.0	98.9	5.0	87.1	8.7	79.0	4.6
FOSA	90.3	3.2	91.3	6.6	73.4	14.2	68.8	3.3
FOSAA	94.5	1.8	81.9	5.1	71.9	14.9	68.7	5.1
N-MeFOSAA	90.6	3.7	89.4	4.1	76.5	9.8	72.6	1.2
N-EtFOSAA	91.3	3.9	87.5	4.1	75.7	16.9	74.0	5.6
MeFBSA	13.4	5.8	124.6	7.4	35.3	4.8	44.5	3.8
MePFHxSA	25.9	3.1	97.0	1.7	35.3	6.2	38.2	0.7
N-MeFOSA	35.2	4.7	88.1	6.0	39.2	9.5	41.8	5.6
N-EtFOSA	30.7	0.3	81.9	3.9	38.1	2.4	44.4	2.6
ADONA	93.5	4.0	104.2	4.7	88.9	9.6	85.5	6.5
HFPO-DA	103.3	6.3	91.2	6.8	93.0	19.3	78.8	9.3
3,6-PFOHpA	101.8	4.8	104.5	9.7	92.4	12.7	90.3	13.1
PF4OPeA	98.4	2.9	102.1	2.8	90.1	1.8	78.5	3.5
PFEESA	98.7	2.9	101.1	4.9	91.0	10.7	87.8	8.8
6:2 Cl-PFESA	95.2	4.2	96.3	4.1	85.5	15.1	79.7	5.8
8:2 Cl-PFESA	90.3	5.1	87.8	6.3	78.5	16.3	76.7	4.7
PFMeSI	99.1	4.4	100.4	5.2	91.9	11.0	87.3	8.7
PFEtSI	98.2	3.5	99.6	5.2	92.5	9.5	85.6	9.1

PFBSI	93.6	5.0	92.1	6.5	77.4	18.2	75.4	5.5
PFECHS	97.7	3.9	100.8	4.8	93.0	11.6	87.6	8.3
5:3 FTB	100.6	4.5	87.8	14.4	106.6	22.5	78.6	18.3
AP-FHxSA	100.3	4.2	104.2	8.4	90.5	5.5	81.8	6.4
TAmP-FHxSA	102.9	5.2	105.0	9.0	98.1	1.5	61.0	27.0
6:2 FTAB	97.6	8.1	103.4	9.8	89.2	3.1	77.1	4.2
FSI	100.7	4.5	64.6	2.1	77.3	4.6	59.2	11.2
PF6	96.3	3.0	85.4	5.2	82.4	7.0	77.3	8.9
BF4	17.3	2.6	6.3	1.1	39.1	17.2	52.9	7.7

Table S13. Instrumental matrix effects of multi-sorbent SPE and WAX-SPE in influent and effluent wastewater. Average and standard deviation is presented and is based on triplicate samples.

	msSPE-effluent		WAX-SPE-effluent		msSPE-influent		WAX-SPE-influent	
	ME (%)	SD	ME (%)	SD	ME (%)	SD	ME (%)	SD
Rosuvastatin	130.8	18.6	130.9	10.0	120.2	11.8	138.0	11.3
Citalopram	61.6	1.0	62.3	2.0	24.5	2.2	32.7	3.5
Efavirenz	167.3	15.1	160.1	9.3	180.6	12.6	192.8	10.5
Ezetimibe	127.1	11.3	130.8	7.9	120.1	8.3	111.5	2.8
Atorvastatin	46.8	3.3	22.3	0.7	47.9	2.3	23.9	1.1
Ticagrelor	131.6	10.1	119.0	4.7	113.5	5.4	122.7	6.9
Sitagliptin	163.1	16.4	153.9	8.8	81.8	4.8	126.9	9.6
Bicalutamide	137.7	11.1	134.9	7.3	138.7	11.1	152.0	8.4
Fluconazole	100.2	2.4	154.9	8.7	64.4	4.8	129.4	8.9
Flecainide	140.5	10.8	140.2	7.8	128.0	6.5	121.1	4.8
Flucloxacillin	138.1	16.0	109.9	5.1	46.1	26.5	NA	NA
Fluoxetine	73.1	5.0	45.9	2.4	30.5	13.8	NA	NA
Bendroflumethiazide	131.6	9.9	171.6	8.6	114.5	5.4	167.5	8.3
5-fluorouracil	36.7	1.9	44.9	1.4	35.6	2.9	42.2	1.6
Efavirenz 8-hydroxy	214.7	6.5	277.4	11.0	279.5	33.2	393.7	14.3
Citalopram desmethyl	50.3	0.8	50.5	0.7	15.8	1.8	20.5	2.6
Celecoxib carboxylic acid	146.3	19.9	140.9	5.4	117.3	10.5	139.9	13.7
Atorvastatin 4-hydroxy	46.3	1.4	17.6	1.1	38.7	3.0	18.4	1.1
Ticagrelor deshydroxyethoxy	124.8	8.2	115.4	7.0	108.0	7.1	123.1	8.7
Seproxetine	78.9	7.5	53.1	2.9	34.0	17.1	NA	NA
Enzalutamide carboxylic acid	105.9	7.9	113.7	4.4	95.4	6.1	104.2	6.2
Sitagliptin N-sulfate	210.2	33.9	230.3	10.8	246.8	24.6	270.9	16.7
Celecoxib	111.2	2.8	60.9	2.6	76.9	5.6	NA	NA
Ciprofloxacin	74.5	5.1	125.3	6.7	23.1	1.1	83.7	5.0
Emtricitabine	98.9	5.4	133.1	5.7	73.3	7.0	145.9	5.5
Rufinamide	69.7	2.7	79.3	4.9	57.8	5.3	81.9	3.0
Rufinamide acid	120.4	7.9	149.6	9.7	67.6	5.4	124.9	4.4
Flecainide meta-o-dealkylated	86.0	2.9	85.8	5.5	92.7	6.3	97.7	3.5
Diflufenican	64.0	10.8	80.8	15.6	31.7	2.6	41.2	6.1
Fluroxipyr	425.3	94.1	381.9	13.1	377.7	85.6	380.3	30.6
Fipronil	151.8	15.3	144.8	8.0	145.6	4.2	147.5	5.5
Fipronil sulfone	162.7	12.7	159.9	7.8	154.3	2.4	153.6	10.5
Benzovindiflupyr	73.9	1.9	71.7	1.3	45.4	2.0	45.6	4.7
Fluopyram	81.0	3.1	54.4	1.0	48.2	15.6	NA	NA
Fluxapyroxad	151.0	13.8	143.5	6.3	144.8	7.7	145.7	11.8

Flufenacet	83.1	3.4	40.9	1.4	48.5	9.8	NA	NA
Fludioxonil	84.4	2.4	53.7	18.1	50.5	16.5	24.5	3.6
Bisphenol AF	159.2	11.7	161.4	1.0	136.8	6.5	134.4	3.7
TFMS	266.5	26.1	216.8	17.1	235.7	23.4	237.9	20.3
PFEtS	216.4	18.6	208.0	4.0	141.4	8.3	126.0	6.5
PFPrS	224.5	51.0	214.6	16.4	251.2	32.1	221.6	26.3
PFBS	97.2	5.9	88.9	4.4	89.0	1.8	89.3	3.0
PFPeS	120.0	8.8	115.1	5.4	109.8	1.3	113.5	3.5
PFHxS	137.4	11.0	132.1	6.6	119.6	3.5	120.6	4.2
PFHpS	140.2	12.1	141.5	7.3	129.2	6.5	140.0	5.6
PFOS	131.0	11.6	122.8	2.7	120.6	5.9	119.3	6.6
PFNS	135.7	7.1	138.4	5.2	125.2	5.6	136.7	6.2
PFDS	135.7	6.8	136.9	4.7	115.1	0.1	121.4	2.5
PFUnDS	136.9	7.3	132.3	2.4	103.2	3.7	121.3	5.7
PFDoDS	136.5	12.3	145.3	6.8	85.4	1.3	126.7	8.9
PFTrDS	115.0	14.1	141.8	12.8	51.3	7.1	126.4	3.7
TFA	133.7	12.8	107.0	0.5	140.5	8.4	132.7	3.5
PFPrA	133.7	11.7	126.5	5.4	125.7	8.3	113.1	8.8
PFBA	40.0	1.5	73.5	1.6	68.2	9.0	64.2	2.8
PFPeA	61.0	2.1	59.4	1.9	66.8	1.2	52.4	5.5
PFHxA	90.3	5.2	85.6	6.2	73.6	1.6	75.2	2.0
PFHpA	110.1	5.7	109.1	3.6	82.3	0.8	85.3	2.3
PFOA	136.7	8.4	146.9	5.5	125.0	4.7	141.9	3.9
PFNA	117.8	8.2	103.2	5.5	111.6	6.3	122.9	4.0
PFDA	201.1	15.3	204.7	6.4	184.8	4.3	195.5	6.1
PFUnDA	146.9	12.3	149.2	7.7	127.0	4.1	137.0	6.1
PFDoDA	200.9	13.6	199.7	4.1	130.1	7.0	137.7	7.2
PFTrDA	196.8	12.5	201.3	9.0	124.7	5.2	157.5	2.8
PFTDA	171.5	9.3	208.8	11.2	105.1	10.9	184.4	6.4
PFHxDA	103.4	5.3	247.9	15.4	19.8	6.6	152.3	4.6
PFOcDA	84.6	4.3	107.9	44.7	4.1	1.4	93.0	16.2
3_3_FTCA	91.3	6.0	93.0	4.6	95.9	2.5	96.0	4.1
5_3 FTCA	102.8	4.8	100.3	3.0	96.4	8.9	106.7	4.2
7_3FTCA	152.0	9.0	164.6	8.6	141.1	3.6	176.5	6.6
6_2 FTUCA	127.0	4.8	132.9	6.9	119.8	3.1	113.7	7.6
8_2_FTUCA	164.0	10.2	181.6	7.0	167.1	4.7	214.3	5.7
10_2_FTUCA	222.4	18.1	226.3	10.6	191.7	3.4	206.7	9.8
4:2 FTSA	489.7	38.8	464.6	12.8	390.4	1.2	382.7	17.3
6:2 FTSA	803.8	71.4	768.1	48.5	808.1	33.1	825.3	22.3
8:2 FTSA	425.3	34.5	421.8	20.1	523.6	23.4	535.5	12.4
10:2 FTSA	464.9	47.8	445.5	36.6	525.2	24.0	627.4	46.1
6:2 diPAP	238.7	15.7	260.9	8.8	373.6	0.9	389.5	19.9
8:2 diPAP	255.7	32.4	293.1	15.8	319.4	12.4	333.2	17.9
6:2-8:2 diPAP	347.6	14.9	388.9	23.1	313.4	2.1	333.9	13.1
SAmPAP	80.1	4.6	100.3	2.2	64.6	2.9	79.7	8.6
diSAmPAP	177.1	7.9	193.5	10.8	146.7	1.6	158.9	2.2

PFHxPA	122.9	5.3	125.7	2.0	99.3	7.8	91.3	12.0
PFOPA	111.5	2.9	119.2	5.7	92.5	3.2	95.7	5.3
6:6 PFPiA	165.7	15.8	166.1	4.0	158.5	3.7	169.3	4.5
6:8 PFPiA	225.5	10.5	240.1	13.8	211.6	5.5	237.0	7.3
8:8 PFPiA	279.4	16.4	337.8	18.3	219.8	17.6	344.5	14.4
FBSA	159.6	14.8	154.3	6.5	136.4	0.8	146.0	4.0
PFHxSA	106.7	6.4	106.9	4.5	90.2	0.7	88.2	3.3
PFOSA	114.8	7.2	113.6	2.5	83.9	2.7	92.3	5.6
FOSAA	193.7	7.2	230.6	11.6	166.3	2.9	171.3	7.7
N-MeFOSAA	98.0	5.9	105.1	3.7	63.6	3.9	67.8	5.2
N-EtFOSAA	124.9	7.5	134.1	3.4	114.3	3.2	122.6	1.3
MeFBSA	125.2	28.3	106.3	6.9	124.3	7.1	138.2	18.2
MePFHxSA	88.5	13.3	84.9	6.8	74.1	4.8	76.6	8.5
N-MeFOSA	103.8	8.2	92.2	13.9	52.7	4.7	53.6	5.7
N-EtFOSA	111.1	11.8	103.8	3.7	48.4	8.2	53.6	9.2
ADONA	121.1	11.0	119.0	8.7	121.4	4.3	124.0	3.6
HFPO-DA	339.4	37.7	331.5	34.8	271.9	23.8	276.2	19.0
3,6-PFOHpA	257.8	29.4	233.4	11.4	322.7	19.7	321.9	14.9
PF4OPeA	73.5	3.1	81.0	3.8	56.9	2.3	62.0	5.2
PFEESA	103.1	7.5	96.7	5.0	97.1	3.7	96.5	3.3
6:2 Cl-PFESA	159.1	15.8	160.7	9.5	154.0	7.6	172.6	5.5
8:2 Cl-PFESA	139.5	11.5	137.4	5.1	108.8	1.6	123.3	5.7
PFMeSI	98.0	8.1	99.4	3.9	110.4	3.4	113.5	4.8
PFEtSI	115.6	8.9	112.2	5.1	105.0	2.5	107.0	4.1
PFBSI	128.9	13.8	126.2	5.4	123.5	5.6	138.4	4.2
PFECHS	132.2	9.3	132.1	5.1	117.7	2.6	125.2	4.5
5:3 FTB	75.9	4.1	40.2	2.5	20.1	11.1	NA	NA
AP-FHxSA	103.1	4.5	68.0	2.5	72.7	6.5	NA	NA
TAmP-FHxSA	94.8	1.2	54.8	1.5	56.4	7.4	NA	NA
6:2 FTAB	119.4	9.9	83.8	5.4	96.8	7.1	NA	NA
FSI	104.3	7.4	112.4	6.8	109.6	9.8	107.4	2.4
PF6	130.1	4.1	121.0	4.0	129.7	12.7	122.2	5.6
BF4	377.3	23.5	139.9	0.9	227.8	24.1	151.4	13.8

*NA: not applicable; data is missing.

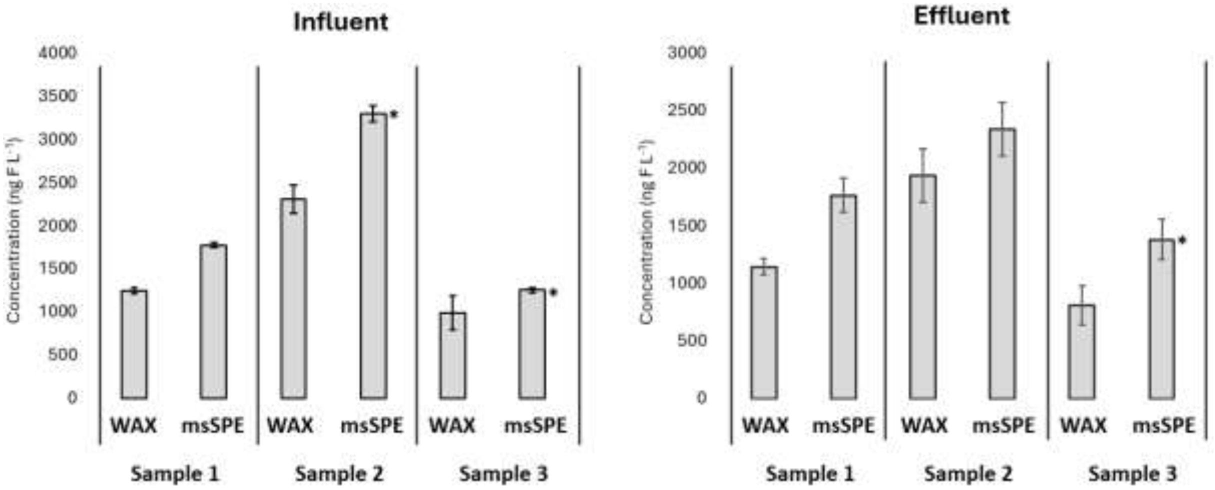

*Figure S3. Concentration of EOF measured by WAX and multi-sorbent SPE. Error bars display standard deviation of triplicate or duplicate extractions (denoted with *).*

References

(1) Horowitz, A. J.; Lum, K. R.; Garbarino, J. R.; Hall, G. E. M.; Lemieux, C.; Demas, C. R. Problems Associated with Using Filtration To Define Dissolved Trace Element Concentrations in Natural Water Samples. *Environmental Science & Technology* **1996**, *30* (3), 954-963. DOI: 10.1021/es950407h.

(2) Aro, R.; Eriksson, U.; Kärrman, A.; Chen, F.; Wang, T.; Yeung, L. W. Y. Fluorine Mass Balance Analysis of Effluent and Sludge from Nordic Countries. *ACS ES&T Water* **2021**, *1* (9), 2087-2096. DOI: 10.1021/acsestwater.1c00168.

(3) Gago-Ferrero, P.; Bletsou, A. A.; Damalas, D. E.; Aalizadeh, R.; Alygizakis, N. A.; Singer, H. P.; Hollender, J.; Thomaidis, N. S. Wide-scope target screening of >2000 emerging contaminants in wastewater samples with UPLC-Q-ToF-HRMS/MS and smart evaluation of its performance through the validation of 195 selected representative analytes. *J Hazard Mater* **2020**, *387*, 121712. DOI: 10.1016/j.jhazmat.2019.121712.

(4) Yeung, L. W. Y.; Stadey, C.; Mabury, S. A. Simultaneous analysis of perfluoroalkyl and polyfluoroalkyl substances including ultrashort-chain C2 and C3 compounds in rain and river water samples by ultra performance convergence chromatography. *Journal of Chromatography A* **2017**, *1522*, 78-85. DOI: 10.1016/j.chroma.2017.09.049.

(5) Karpecki, P. M.; Sheppard, J. D. Perfluorohexyloctane ophthalmic solution: a review of a prescription treatment for dry eye disease that directly targets tear evaporation. *Expert Review of Ophthalmology* **2023**, *18* (6), 355-364. DOI: 10.1080/17469899.2023.2275586.

(6) Houtz, E. F.; Sedlak, D. L. Oxidative Conversion as a Means of Detecting Precursors to Perfluoroalkyl Acids in Urban Runoff. *Environmental Science & Technology* **2012**, *46* (17), 9342-9349. DOI: 10.1021/es302274g.

(7) Swedish National Board of Health and Welfare. Statistikdatabas för läkemedel. Sitagliptin. https://sdb.socialstyrelsen.se/if_lak/val.aspx (accessed 2025-07-02).

(8) Alba, M.; Sheng, D.; Guan, Y.; Williams-Herman, D.; Larson, P.; Sachs, J. R.; Thornberry, N.; Herman, G.; Kaufman, K. D.; Goldstein, B. J. Sitagliptin 100 mg daily effect on DPP-4 inhibition and compound-specific glycemic improvement. *Curr Med Res Opin* **2009**, *25* (10), 2507-2514. DOI: 10.1185/03007990903209514.

(9) Inoue, M.; Sumii, Y.; Shibata, N. Contribution of Organofluorine Compounds to Pharmaceuticals. *ACS Omega* **2020**, *5* (19), 10633-10640. DOI: 10.1021/acsomega.0c00830.

(10) Swedish National Board of Health and Welfare. Statistikdatabas för läkemedel. https://sdb.socialstyrelsen.se/if_lak/val.aspx. (accessed 2026-01-14).

(11) Swedish Medical Product agency. Sök läkemedelsfakta. https://www.lakemedelsverket.se/sv/sok-lakemedelsfakta. (accessed 2024-05-03).

(12) S111 | PMTPFAS | Fluorine-containing Compounds in PMT Suspect Lists. (accessed 2025-07-01).

(13) Luo, Y. S.; Aly, N. A.; McCord, J.; Strynar, M. J.; Chiu, W. A.; Dodds, J. N.; Baker, E. S.; Rusyn, I. Rapid Characterization of Emerging Per- and Polyfluoroalkyl Substances in Aqueous Film-Forming Foams Using Ion Mobility Spectrometry-Mass Spectrometry. *Environ Sci Technol* **2020**, *54* (23), 15024-15034. DOI: 10.1021/acs.est.0c04798.
(14) Menger, F.; Boström, G.; Jonsson, O.; Ahrens, L.; Wiberg, K.; Kreuger, J.; Gago-Ferrero, P. Identification of Pesticide Transformation Products in Surface Water Using Suspect Screening Combined with National Monitoring Data. *Environmental Science & Technology* **2021**, *55* (15), 10343-10353. DOI: 10.1021/acs.est.1c00466.
(15) Newton, S.; McMahen, R.; Stoeckel, J. A.; Chislock, M.; Lindstrom, A.; Strynar, M. Novel Polyfluorinated Compounds Identified Using High Resolution Mass Spectrometry Downstream of Manufacturing Facilities near Decatur, Alabama. *Environ Sci Technol* **2017**, *51* (3), 1544-1552. DOI: 10.1021/acs.est.6b05330.
(16) Buck, R. C.; Franklin, J.; Berger, U.; Conder, J. M.; Cousins, I. T.; de Voogt, P.; Jensen, A. A.; Kannan, K.; Mabury, S. A.; van Leeuwen, S. P. Perfluoroalkyl and polyfluoroalkyl substances in the environment: terminology, classification, and origins. *Integr Environ Assess Manag* **2011**, *7* (4), 513-541. DOI: 10.1002/ieam.258.
(17) Lange, C. C. Anaerobic biotransformation of N-methyl perfluorobutanesulfonamido ethanol and N-ethyl perfluorooctanesulfonamido ethanol. *Environmental Toxicology and Chemistry* **2017**, *37* (3), 768-779. DOI: 10.1002/etc.4014.
(18) Cockshott, I. D. Bicalutamide. *Clinical Pharmacokinetics* **2004**, *43* (13), 855-878. DOI: 10.2165/00003088-200443130-00003.
(19) Zhang, J.; Liu, Z. H.; Zhong, S. S.; Wang, H.; Caidan, B.; Yin, H.; Dang, Z. Strategy for effective inhibition of arylsulfatase/beta-glucuronidase to prevent deconjugation of sulfate and glucuronide conjugates in wastewater during sample collection and storage. *Sci Total Environ* **2020**, *703*, 135536. DOI: 10.1016/j.scitotenv.2019.135536.
(20) Henning, N.; Falas, P.; Castronovo, S.; Jewell, K. S.; Bester, K.; Ternes, T. A.; Wick, A. Biological transformation of fexofenadine and sitagliptin by carrier-attached biomass and suspended sludge from a hybrid moving bed biofilm reactor. *Water Res* **2019**, *167*, 115034. DOI: 10.1016/j.watres.2019.115034.
(21) Massbank.eu. N-acetyl Sitagliptin. MSBNK-BAFG-CSL2311091195. . (accessed 2025-07-01).
(22) Kärrman, A.; Wang, T.; Kallenborn, R.; Langseter, A. M.; Grønhovd, S. M.; Ræder, E. M.; Lyche, J. L.; Yeung, L.; Chen, F.; Eriksson, U.; Aro, R.; Fredriksson, F. *PFASs in the Nordic environment - Screening of Poly- and Perfluoroalkyl Substances (PFASs) and Extractable Organic Fluorine (EOF) in the Nordic Environment*; Nordic Council of Ministers, 2019.
(23) Miyake, Y.; Yamashita, N.; Rostkowski, P.; So, M. K.; Taniyasu, S.; Lam, P. K. S.; Kannan, K. Determination of trace levels of total fluorine in water using combustion ion chromatography for fluorine: A mass balance approach to determine individual perfluorinated chemicals in water. *Journal of Chromatography A* **2007**, *1143* (1-2), 98-104. DOI: 10.1016/j.chroma.2006.12.071.
(24) Jiao, E.; Larsson, P.; Wang, Q.; Zhu, Z.; Yin, D.; Karrman, A.; van Hees, P.; Karlsson, P.; Qiu, Y.; Yeung, L. W. Y. Further Insight into Extractable (Organo)fluorine Mass Balance Analysis of Tap Water from Shanghai, China. *Environ Sci Technol* **2023**, *57* (38), 14330-14339. DOI: 10.1021/acs.est.3c02718.
